# Oncostatin M induced by STAT5-activating oncogenes promotes disease progression in hematologic malignancies

**DOI:** 10.1038/s41392-025-02491-6

**Published:** 2025-12-11

**Authors:** Michael Rassner, Tony Andreas Müller, Kirstyn Anne Crossley, Geoffroy Andrieux, Sabina Schaberg, Cornelia Endres, Lena Jakob, Teresa Poggio, Natalie Köhler, Julia Kolter, Gerhard Müller-Newen, Katharina Schönberger, Nina Cabezas-Wallscheid, Irene Gonzalez-Menendez, Leticia Quintanilla-Martinez, Melissa Zwick, Driti Ashok, Tanja Nicole Hartmann, Olaf Groß, Oliver Gorka, Marie Follo, Anna Lena Illert, Melanie Boerries, Robert Zeiser, Justus Duyster

**Affiliations:** 1 Department of Medicine I, Medical Center – University of Freiburg, Faculty of Medicine, University of Freiburg, Freiburg, Germany; 2 Department I of Internal Medicine, Center for Integrated Oncology, Aachen-Bonn-Cologne-Duesseldorf, University of Cologne, Cologne, Germany; 3 Faculty of Biology, University of Freiburg, Freiburg, Germany; 4 Institute of Medical Bioinformatics and Systems Medicine, Medical Center – University of Freiburg, Faculty of Medicine, University of Freiburg, Freiburg, Germany; 5 CIBSS - Centre for Integrative Biological Signaling Studies, University of Freiburg, Freiburg, Germany; 6 Institute for Immunodeficiency, Center for Chronic Immunodeficiency (CCI), Medical Center – University of Freiburg, Faculty of Medicine, University of Freiburg, Freiburg, Germany; 7 Institute for Infection Prevention and Control, Medical Center - University of Freiburg, Faculty of Medicine, University of Freiburg, Freiburg, Germany; 8 Institute of Biochemistry and Molecular Biology, RWTH Aachen University, Aachen, Germany; 9 Max Planck Institute of Immunobiology and Epigenetics, Freiburg, Germany; 10 Department of Pathology and Neuropathology, University Hospital Tübingen & Comprehensive Cancer Center Tübingen, Tübingen, Germany; 11 Cluster of Excellence iFIT (EXC 2180) “Image-Guided and Functionally Instructed Tumor Therapies”, Eberhard-Karls University of Tübingen, Tübingen, Germany; 12 Institute of Neuropathology, Medical Center – University of Freiburg, Faculty of Medicine, University of Freiburg, Freiburg, Germany; 13 German Cancer Consortium (DKTK), Partner site Freiburg, a partnership between DKFZ and Medical Center - University of Freiburg, Freiburg, Germany; 14 Medical Department for Hematology and Oncology, Klinikum Rechts der Isar, Technische Universität München, München, Germany; 15 Department of Hematology and Medical Oncology, University Medical Center Göttingen (UMG), Göttingen, Germany

**Keywords:** Cancer microenvironment, Haematological cancer, Haematological cancer, Tumour immunology, Drug development

## Abstract

Understanding the interplay between oncogenic mutations and the tumor microenvironment could help improve therapy for hematological malignancies. We found that the STAT5-activating oncogenes JAK2 p.V617F, FLT3-ITD, and BCR::ABL1 induce oncostatin M (OSM), which triggers disease progression and immunosuppression. The OSM receptor was predominantly expressed on nonhematopoietic bone marrow (BM) stromal cells. OSM reprogrammed these cells via STAT3 and induced the secretion of cytokines connected to T-cell exhaustion, including IL-6 and MCP-1. Compared with control mice, OSM-overexpressing mice presented reduced T-cell numbers, increased levels of inhibitory receptors on T cells, and elevated lactic acid production by BM stromal cells. OSM induced the expansion of myeloid cells which suppressed T cells. Conversely, genetic deletion of *Osm* in a JAK2 p.V617F-driven polycythemia vera mouse model reduced polycythemia, BM fibrosis, inflammatory cytokine levels and the expression of inhibitory markers on T cells. Transcriptomic analyses of T cells from OSM-overexpressing mice revealed enrichment of IL6–JAK–STAT3 and inflammatory signaling pathways. Additionally, pharmacological inhibition of OSM reduced disease activity and cytokine production. These findings establish OSM as a key mediator linking oncogenic STAT5 activation to remodeling of the microenvironment and immune suppression. Targeting OSM signaling therefore represents a promising therapeutic strategy to alleviate disease progression in myeloproliferative neoplasms and related malignancies.

## Introduction

A link between oncogenic signaling and the tumor microenvironment (TME) has been demonstrated in several hematological malignancies.^1–3^ Aberrant activation of signaling pathways such as JAK–STAT, FLT3, and BCR::ABL1 not only drives uncontrolled cell proliferation but also remodels the surrounding stroma to create a supportive niche for malignant hematopoiesis. Among these pathways, activation of signal transducer and activator of transcription 5 (STAT5) has emerged as a central node in myeloproliferative neoplasms (MPNs) and acute myeloid leukemia (AML). MPNs are clonal hematopoietic stem cell disorders characterized by the sustained proliferation of one or more myeloid lineages, leading to conditions such as polycythemia vera (PV), essential thrombocythemia (ET), and primary myelofibrosis (PMF).^4^ We previously demonstrated that oncogenic FLT3-ITD causes STAT5 activation, thereby promoting MPNs in mice.^5^ Upon activation, STAT5 induces a distinct transcriptional program encompassing several cytokines and growth factors, notably the interleukin-6 (IL-6) family cytokine oncostatin M (OSM).^6,7^ Increased OSM expression has been observed in both patients and mouse models harboring mutations commonly associated with myeloid malignancies, including JAK2 p.V617F, KIT p.D816V, FIP1L1::PDGFRA, and FLT3-ITD.^5,7–9^ Transplantation of bone marrow (BM) cells overexpressing OSM into irradiated mice results in a lethal myeloproliferative disease characterized by the expansion of both OSM-expressing and non-OSM-expressing cells.^5,10^ In our previous study, ectopic OSM expression in a FLT3-TKD p.D835Y mouse model, which alone does not activate STAT5, shifted the disease phenotype from lymphoid to myeloproliferative and significantly shortened survival, closely resembling the more aggressive FLT3-ITD phenotype.^5^ Notably, in newly diagnosed AML patients, high circulating levels of mesenchymal stromal cell-derived OSM receptor (OSMR) have been identified as the strongest negative prognostic marker, with OSMR^high^ patients exhibiting elevated serum OSM levels.^11^ Despite these associations, the biological role of OSM/OSMR signaling in leukemogenesis and disease progression has remained poorly understood.

OSM was originally found as an inhibitor of melanoma cell proliferation in vitro, but subsequent studies revealed its context-dependent and predominantly proinflammatory role in vivo. As a member of the IL-6 cytokine family, OSM signals through a heterodimeric receptor complex composed of gp130 and either LIFRβ (OSMR type I) or OMSR (OSMR type II). In mice, LIF signals through the type I receptor, whereas murine OSM signals primarily via the type II receptor.^12–14^ OSM strongly induces cytokines and chemokines, including IL-6, MCP-1/CCL2, and CXCL12 in fibroblasts and stromal cells.^7,15–23^ This capacity to modulate cytokine production positions OSM as a potential master regulator of inflammatory crosstalk within the BM niche. In hematologic malignancies, chronic inflammation and cytokine dysregulation are increasingly recognized as key contributors to disease progression, fibrosis, and therapy resistance.^24^ However, most research has focused on downstream cytokines such as IL-6 and TNF-α, while the upstream regulators that initiate and sustain the inflammatory milieu remain incompletely defined.^25,26^ Since OSM can be induced by oncogenic STAT5 signaling and is capable of reprogramming stromal and immune cell function, it may serve as a critical molecular link between oncogene-driven transformation and the establishment of an immunosuppressive TME. Understanding how OSM shapes stromal and immune cell interactions could therefore provide important insights into the mechanisms underlying leukemic progression and identify novel therapeutic targets for MPNs and AML.

Despite evidence linking OSM to myeloid neoplasms, key questions remain regarding its cellular sources and targets, its effects on stromal and immune cell function, and whether OSM inhibition can reduce disease burden. To address these questions, we investigated the induction and functional consequences of OSM signaling in hematologic malignancies driven by STAT5-activating oncogenes. We analyzed OSM expression in cells transformed with JAK2 p.V617F, FLT3-ITD, BCR::ABL1, and NPM1::ALK, and characterized OSMR expression in BM-derived stromal and hematopoietic cells. Functional studies were conducted using murine models of leukemia and PV to assess OSM effects on BM stromal metabolism, cytokine secretion, and immune cell composition. Furthermore, we utilized both genetic (*Osm* knockout mice) and pharmacologic (OSMR fusion protein and anti-OSM antibody) strategies to determine the potential of OSM inhibition.

In this study, we demonstrate that OSM expression is induced by several STAT5-activating oncogenes including JAK2 p.V617F, FLT3-ITD, BCR::ABL1, and NPM1::ALK. We show that OSM acted predominantly on BM stromal cells expressing OSMR, triggering proinflammatory signaling via STAT3 activation and leading to metabolic reprogramming, enhanced cytokine secretion, and recruitment of immunosuppressive myeloid cells. Overexpression of OSM in murine models induced a myeloproliferative phenotype with reduced T-cell numbers, upregulation of inhibitory markers (PD-1, TIM-3) on T cells and increased the expansion of T-cell-suppressive myeloid cells. In a murine model of JAK2 p.V617F-driven PV, genetic deletion or pharmacologic inhibition of OSM reduced polycythemia, spleen size, BM fibrosis and the levels of proinflammatory cytokines, establishing OSM as a critical mediator of oncogene-induced inflammation and immune suppression. These findings uncover a previously unrecognized oncogenic STAT5-OSM axis that drives stromal and immune remodeling in hematologic malignancies. We demonstrate that disrupting OSM signaling attenuates disease in MPN and AML models, establishing OSM as a central mediator linking oncogenic signaling to the inflammatory TME and unveiling a promising therapeutic target.

## Results

### STAT5-activating oncogenes induce OSM in myeloid cells

**Fig. 1:**
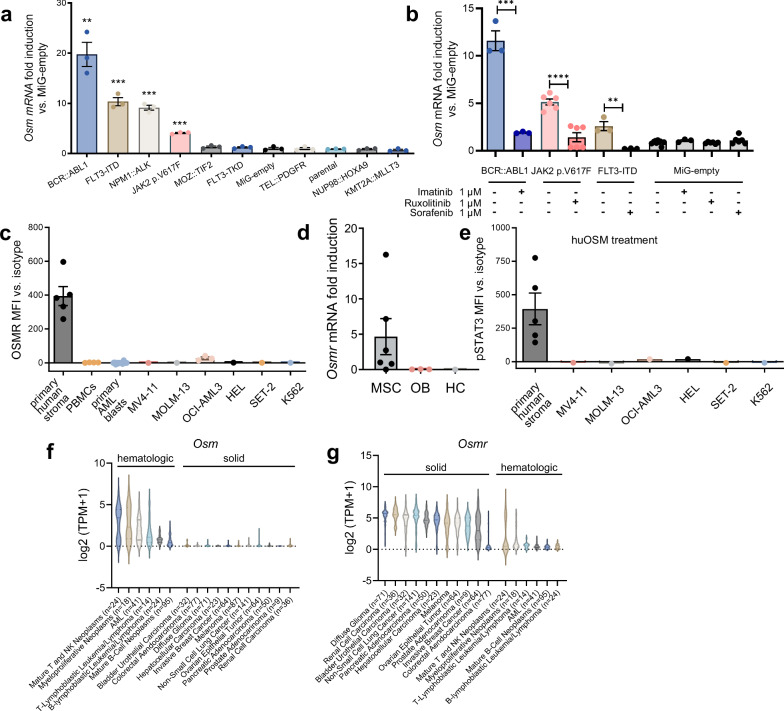
OSM is induced by STAT5-activating oncogenes in a kinase-dependent manner and acts on bone marrow stromal cells via STAT3 phosphorylation. **a** 32D cells were transduced with various oncogenes and starved of cytokines and serum for twelve hours. Quantitative PCR revealed *Osm* upregulation in BCR::ABL1-, FLT3-ITD-, NPM1::ALK-, and JAK2 p.V617F-positive cells. *Osm* expression values were normalized to *Gapdh* and empty vector (MiG-empty) expression values. *N* ≥ 3 experiments; *p* values (compared with MiG-empty cells): ***p < 0.001; **p < 0.01. The data are presented as the means ± SEMs. **b** 32D cells transduced with oncogenes were serum-starved plus inhibitor-treated as indicated for twelve hours. *Osm* expression values were normalized to *Gapdh* and MiG-empty vector expression values. *P* values (compared with MiG-empty cells): ***p < 0.001; **p < 0.01. The data are presented as the means ± SEMs. **c** Flow cytometric analysis of OSMR surface levels in primary human stromal cells, primary AML blasts, and human myeloid leukemic cell lines. The mean fluorescence intensity (MFI) of the isotype control was subtracted from the OSMR MFI values. Each dot represents one measurement. The data are presented as the means ± SEMs. **d** Primary murine hematopoietic BM cells (HCs) did not express *Osmr* transcripts after 40 PCR cycles, whereas mesenchymal stromal cell (MSC) populations expressed *Osmr*. MSC = mesenchymal stromal cells, OB = osteoblasts, ND = not detected. Each dot represents one measurement. The data are presented as the means ± SEMs. **e** Flow cytometric analysis of pSTAT3 levels in primary human stromal cells and human myeloid leukemic cell lines after huOSM treatment (2.5 h, 10 ng/mL). The mean fluorescence intensity (MFI) of the isotype control was subtracted from the pSTAT3 antibody MFI values. Each dot represents one measurement. The data are presented as the means ± SEMs. **f**, **g** *OSM* (**f**) and *OSMR* (**g**) expression across hematological and solid neoplasms utilizing Expression Public 24Q2 and lineage subtype grouping on the DepMap portal (https://depmap.org)

To explore whether *Osm* expression is induced by STAT5-activating oncogenes, we transduced 32D cells with different oncogenes. These included tyrosine kinase mutants known to activate STAT5, such as JAK2 p.V617F, FLT3-ITD, BCR::ABL1, or NPM1::ALK, as well as oncogenes that do not activate STAT5, including FLT3-TKD,^5^ MOZ::TIF2, NUP98::HOXA9, or KMT2A::MLLT3 (supplementary Fig. 1a). We observed elevated expression of *Osm* in 32D cells transduced with STAT5-activating oncogenes compared with mock-transfected cells (empty vector) or parental cells (Fig. 1a). Conversely, cells transduced with oncogenes that do not activate STAT5 did not exhibit increased *Osm* expression (Fig. 1a). To validate whether *Osm* expression depends on oncogenic kinase activity, we treated JAK2 p.V617F^+^, FLT3-ITD^+^, or BCR::ABL1^+^, cells with the kinase inhibitors (KIs) imatinib, ruxolitinib, and sorafenib. Treatment with these KIs abrogated the increase in *Osm* expression caused by the mutant kinases (Fig. 1b). Changes in *Osm* mRNA levels paralleled OSM protein production, as determined by ELISA of cell culture supernatants expressing STAT5-activating oncogenes (supplementary Fig. 1b, c). These findings link the STAT5-activating oncogenes that are frequently found in myeloid neoplasms to *Osm* transcription.

### The OSM receptor is expressed by BM stromal cells

To characterize which cells respond to OSM produced by oncogene-transduced myeloid cells, we conducted flow cytometry-based analyses to assess the surface expression of the OSM receptor (OSMR) on primary human stromal and hematopoietic cells. Our findings revealed strong surface expression of OSMR on primary human BM stromal cells derived from five different donors. We did not observe significant receptor levels on the surface of peripheral blood mononuclear cells (PBMCs) or CD45^+^TCRαβ^+^ T cells from healthy donors, primary AML patient blasts, or human AML cell lines (MV4-11, MOLM-13, OCI-AML3, HEL), an essential thrombocythemia (ET) cell line (SET-2), or a CML cell line (K562) (Fig. 1c, supplementary Fig. 2). To identify the stromal cell population expressing OSMR, we sorted different murine stromal cell populations. We observed no *Osmr* transcripts in primary murine hematopoietic cells (HCs) or osteoblasts (OBs), whereas mesenchymal stromal cells (MSCs) expressed *Osmr* (Fig. 1d). To investigate whether OSMR downstream signaling in BM stromal cells is induced upon OSM stimulation, we studied the activation of STAT3, a key downstream effector of OSMR signaling.^23,27^ We observed STAT3 phosphorylation in human primary stromal cells from five different donors following OSM treatment (Fig. 1e). Conversely, the AML, ET, and CML cell lines did not display pSTAT3 signaling (Fig. 1e). Our findings are in line with recent reports where *Osmr* mRNA was undetectable in BM myeloid cells and HSPCs^11,16^ but abundantly expressed by endothelial cells and mesenchymal stromal cells.^11,16,28–30^

To investigate the expression of OSM and OSMR beyond BM cells, we conducted a comprehensive data analysis via the DepMap portal. The data derived from the repository revealed that OSM expression is predominantly restricted to hematologic malignancies, whereas OSMR expression is primarily observed in solid tumors (Fig. 1f, g).

These data indicate that hematopoietic cells produce OSM, whereas nonhematopoietic BM stromal cells can respond to OSM on the basis of their OSMR expression.

### OSM is elevated in samples from patients with AML and MPN

**Fig. 2:**
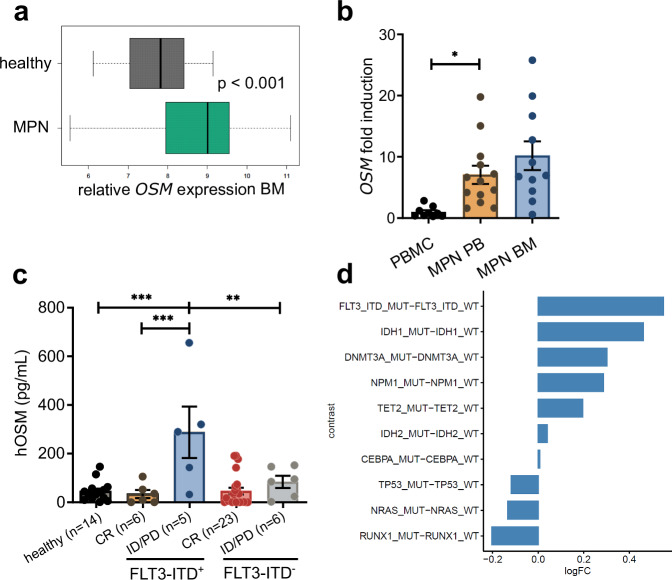
OSM is elevated in patients with MPN and FLT3-ITD^+^ AML. **a** Analysis of relative *OSM* expression in a previously published microarray expression database.^31^ Data are represented as medians with interquartile ranges (IQRs) (25th–75th percentiles) within the box and whiskers extending to the minimum and maximum values within 1.5× IQRs. **b** *OSM* expression in MPN PBMCs (*n* = 13) and BM (*n* = 11). The expression values were normalized to PRDM4 expression and to those of healthy PBMC samples (*n* = 9). *p < 0.05, **p < 0.01. Each dot represents one sample. The data are presented as the means ± SEMs. **c** Levels of human OSM in the plasma of AML patients stratified by the FLT3-ITD status. CR = complete remission, ID = initial diagnosis, PD = progressive disease (blast persistence or relapse). *p < 0.05, **p < 0.01, ****p < 0.001. One-way ANOVA with posttest correction for multiple comparisons. Each dot represents one sample. The data are presented as the means ± SEMs. **d** Differential expression analysis for *OSM* transcript (log2-fold change) samples from the BeatAML cohort.^32^ Samples were selected for “Initial Acute Leukemia Diagnosis” → “Bone Marrow Aspirate” and displayed genes for “mutant” vs. “WT”

To assess OSM levels in patients with hematologic malignancies, we reanalyzed data from a publicly available dataset^,^^31^ which revealed significantly higher *OSM* expression in MPN samples than in healthy control samples (Fig. 2a). We detected higher relative *OSM* expression in the peripheral blood (PB) and BM isolated from patients with MPN than in those from healthy controls (Fig. 2b; supplementary Table 1). Additionally, we assessed OSM levels in the plasma of AML patients and observed substantially higher levels in patients with FLT3-ITD^+^ AML and active disease (i.e., initial diagnosis, blast persistence, or relapse) than in FLT3-ITD^+^ patients in remission or in FLT3-ITD^-^ patients (Fig. 2c; supplementary Table 2). Furthermore, analysis of the BeatAML cohort^32^ revealed the highest fold change in the number of *OSM* transcripts in patients with FLT3-ITD^+^ AML (Fig. 2d). Patients with RUNX1-mutated AML, in which RUNX1 mutually inhibits STAT5,^33^ presented reduced *OSM* transcript levels (Fig. 2d). Examination of the TCGA database further revealed elevated *OSM* expression in AML patients compared with healthy controls, along with a trend toward improved survival among *OSM*^low^ AML patients (supplementary Fig. 3a, b).

### OSM stimulation increases the metabolic activity of BM stroma cells

To understand the functional consequences of OSM signaling within the BM microenvironment, we next investigated how OSM stimulation affects BM stromal cells. Given the expression of OSMR on these nonhematopoietic cells and the established link between STAT5-activating oncogenes and OSM production, we hypothesized that OSM may alter stromal cell behavior, thereby influencing disease progression.

OSM has been implicated in regulating cellular metabolism.^34,35^ Therefore, we first sought to investigate whether OSM treatment of stroma cells could induce metabolic alterations and whether such alterations are JAK1/2 dependent, as OSMR downstream signaling occurs via JAK1/2. The BM stromal cell lines OP9 and embryonic liver-derived EL08-1D2^36^ presented significant increases in both oxidative phosphorylation and glycolytic activity when exposed to OSM (supplementary Fig. 4a–f). To determine whether this effect is dependent on JAK-mediated signaling, we treated OP9 cells with OSM alone, the JAK1/2-specific inhibitor ruxolitinib, or a combination of both. OSM treatment markedly increased mitochondrial activity parameters such as basal respiration, maximal respiration, spare respiratory capacity, and ATP production. Cotreatment with ruxolitinib inhibited the increase in these parameters (supplementary Fig. 4c). OSM treatment also enhanced basic glycolytic activity, glycolytic capacity, and reserve in OP9 cells, whereas cells treated with OSM plus ruxolitinib did not exhibit changes in these parameters (supplementary Fig. 4d). We subsequently conducted a metabolomics analysis to investigate the specific effects of OSM treatment on the abundance of various metabolites. We detected an elevated concentration of LA in the OSM-treated OP9 cells (supplementary Fig. 4g). The release of LA has been previously shown to induce T-cell exhaustion.^37^

### OSM induces the release of proinflammatory cytokines and chemokines in BM stromal cells

**Fig. 3:**
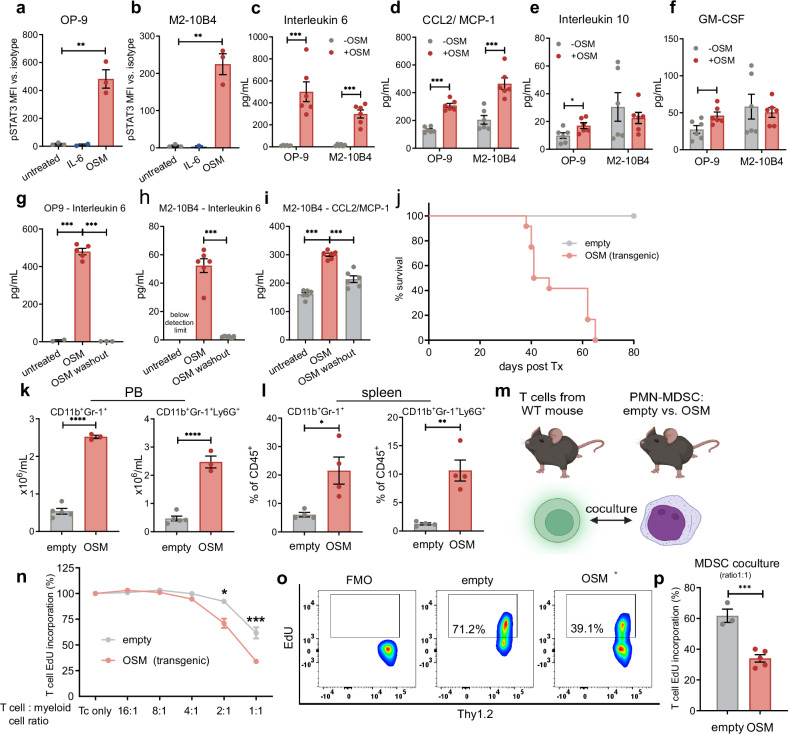
OSM increases the metabolic activity and cytokine and chemokine expression of BM stromal cells and induces the expansion of immunosuppressive myeloid cells. **a**, **b** Flow cytometric analysis of STAT3 phosphorylation (pY705) in OP9 (**a**) or M2-10B4 (**b**) cells after treatment with OSM or IL-6. MFI values were calculated by subtracting the isotype MFI from the pSTAT3 MFI. *N* = 3. The data are presented as the means ± SEMs. **c**–**f** OSM treatment induces the release of IL-6 (**c**) and CCL2/MCP-1 (**d**) into the supernatant of OP9 or M2-10B4 cells and the release of IL-10 (**e**) and GM-CSF (**f**) in OP9 cells. **g**–**i** Washout of OSM reduced the secretion of IL-6 in OSM-pretreated OP9 cells (**g**) and that of IL-6 and CCL2/MCP-1 in M2-10B4 (**h**, **i**) cells. *N* ≥ 5. *p < 0.05, **p < 0.01, ***p < 0.001. The data are presented as the means ± SEMs. **j** OSM overexpression induces a lethal MPN-like disease in a murine BM transplantation model. *N* = 10 vs. 9. **k**, **l** Lineage analysis by flow cytometry of peripheral blood (PB) (**k**) and spleen (**l**) CD45^+^ cells 2 months after BM transplantation. *N* = 3 vs. 5, and 4 vs. 4, *p < 0.05, **p < 0.01, ****p < 0.0001. The data are presented as the means ± SEMs. **m** CD11b^+^Gr-1^+^Ly6G^+^Ly6C^low^ cells at decreasing ratios were cocultured with 2 × 10^5^ CD3^+^/CD28^+^ activated WT T cells. Figure generated with BioRender. **n**–**p** Proliferation of Thy1.2^+^ T cells after coculture (**m**) was assessed by EdU incorporation. *N* = 3 vs. 5. ***p < 0.001. The data are presented as the means ± SEMs

To further explore the effects of OSM on the BM stroma, we used the murine BM stroma cell lines OP9 and M2-10B4 (instead of EL08-1D2, which is derived from the embryonic liver^36^). Like the human BM stroma cell lines, OP9 and M2-10B4 exhibited increased STAT3 phosphorylation upon OSM treatment, which is an indicator of downstream signaling (Fig. 3a, b). We hypothesized that the activation of stroma cells by OSM may elicit indirect effects on the expansion of myeloid cells via the production of proinflammatory cytokines. To address this, we analyzed the production of a set of cytokines in OSM-treated versus untreated murine BM stroma cell lines. In agreement with studies on other stroma cell lines,^23,38^ we detected a strong increase in IL-6 production in OSM-treated OP9 and M2-10B4 cells (Fig. 3c). Furthermore, OSM-treated OP9 and M2-10B4 cells presented significantly elevated production of the monocyte-attracting chemokines MCP-1/CCL2 (Fig. 3d). GM-CSF and IL-10 were upregulated only in OP9 cells upon OSM treatment (Fig. 3e, f).

To confirm that IL-6 and MCP-1 induction was specifically driven by OSM, we performed washout experiments. Removal of OSM after initial stimulation reduced IL-6 levels back to baseline in both OP9 and M2-10B4 cells (Fig. 3g, h) and attenuated MCP-1 production in M2-10B4 cells (Fig. 3i). Additionally, we cocultured human BM stromal cells (HS5) with primary FLT3-ITD^+^ and FLT3-ITD^-^ AML blast cells and measured cytokine transcript levels in human stromal cells. Compared with those cocultured with FLT3-ITD- blasts, HS5 cells cocultured with FLT3-ITD^+^ AML blasts presented higher transcript levels of *CSF2* (GM-CSF), *CCL2* (MCP-1), and IL6 (supplementary Fig. 5a–c). These findings demonstrate that features observed in the mouse model are recapitulated in primary patient samples of myeloid malignancies.

### OSM overexpression promotes the expression of immunosuppressive myeloid cells

On the basis of the metabolic reprogramming of stroma cells, as well as the release of LA and proinflammatory cytokines and chemokines, we hypothesized that OSM may promote the expansion of immunosuppressive myeloid cells. To test this hypothesis, we implemented a murine BM transplantation model in which we overexpressed OSM. We observed that overexpressing OSM in the BM led to the induction of a lethal MPN-like disease (Fig. 3j). The analysis of the myeloid compartment revealed an increase in the proportion of CD11b^+^Gr-1^+^Ly6G^+^Ly6C^low^ cells—in tumor-bearing mice, defined as polymorphonuclear (PMN)-MDSCs as previously described^39^—that were found in the PB and spleens of OSM-overexpressing mice but not in those of WT mice (Fig. 3k, l). To test for immunosuppressive activity, we cocultured these myeloid cells isolated from the spleens of OSM-overexpressing mice with CD3/CD28-activated WT T cells at decreasing ratios (Fig. 3m, n). We observed that T cells cocultured with myeloid cells from OSM-overexpressing animals exhibited reduced proliferation compared with those cocultured with CD11b^+^Gr-1^+^Ly6G^+^Ly6C^low^ cells from empty vector-treated mice (Fig. 3n–p). These findings demonstrate that OSM overexpression in mice induces the expansion of T-cell-suppressed myeloid cells.

### OSM overexpression induces the upregulation of T-cell exhaustion markers

**Fig. 4:**
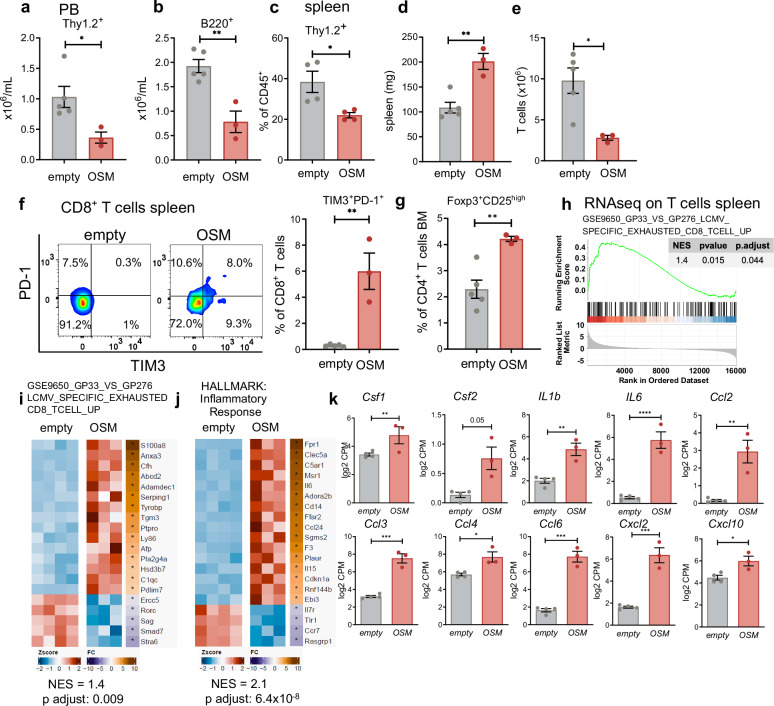
Overexpression of OSM reduces T-cell numbers and induces T-cell exhaustion. **a**–**c** Lineage analysis of peripheral blood (PB) and spleen CD45^+^ cells via flow cytometry. *N* = 5 vs. 3, and 4 vs. 4. *p < 0.05, **p < 0.01. The data are presented as the means ± SEMs. **d** Spleen weights of the mice transplanted with empty vector or OSM-encoding vector BM. *N* = 5 vs. 3. **p < 0.01. The data are presented as the means ± SEMs. **e** Spleen T-cell numbers of mice transplanted with empty vector or OSM-expressing vectors. *N* = 4 vs. 4, *p < 0.05. The data are presented as the means ± SEMs. **f** Flow cytometric analyses of programmed cell death protein 1 (PD-1) and T-cell immunoglobulin and mucin-domain containing-3 (TIM-3) on CD8^+^ T cells isolated from the spleen. *N* = 5 vs. 3. **p < 0.01. **g** Percentage of CD25^+^FOXP3^+^ CD4^+^ T cells from the bone marrow (BM). *N* = 5 vs. 3. **p < 0.01. The data are presented as the means ± SEMs. **h**, **i** Gene set enrichment analyses (**h**) of the transcriptomes of flow cytometry-sorted bulk CD45^+^Thy1.2^+^ T cells from OSM-expressing mice vs. WT T cells. Genes upregulated during exhaustion according to GSE9650^40^ were scored. The top 20 significantly regulated genes in that process are shown as a heatmap (**i**). NES: normalized enrichment score. *N* = 3 vs. 4. **j** Top 20 significantly regulated genes from the HALLMARK “Inflammatory Response”. **k** Depiction of specific transcript levels in T cells from OSM-expressing and control mice. *N* = 4 vs. 3, *p < 0.05, **p < 0.01, ***p < 0.001, ****p < 0.0001. The data are presented as the means ± SEMs. All experiments were conducted on material from mice two months after BM transplantation

To extend our findings to myeloid cells, we next characterized the impact of OSM overexpression on lymphoid cells. In contrast to our findings in myeloid cells, B- and T-cell numbers decreased in the PB and spleen of mice that received BM from donors with constitutive OSM overexpression (Fig. 4a–c). Additionally, despite an increase in the spleen weight (Fig. 4d, e), the absolute number of T cells in the spleen of these animals was reduced. Compared with those from WT mice, T cells from OSM-overexpressing mice presented increased expression of exhaustion markers, including T-cell immunoglobulin and mucin-domain containing-3 (TIM-3) and PD-1 (Fig. 4f), as well as an increased percentage of CD4^+^CD25^+^FoxP3^+^ regulatory T cells (Tregs; Fig. 4g, supplementary Fig. 6a). Additionally, we observed increased surface expression of programmed death-ligand 1 (PD-L1) in various immune cell subtypes isolated from OSM-overexpressing mice (supplementary Fig. 6b). Both OSM-expressing (EGFP^+^) and nonexpressing (EGFP^-^) cells presented increased PD-L1 surface levels (supplementary Fig. 6b).

To study the changes in T cells in more detail, we performed a transcriptomic analysis of T cells from OSM-overexpressing mice. This analysis revealed >2000 differentially expressed genes (adjusted *p* value < 0.05) in T cells isolated from these mice compared with those isolated from WT mice (supplementary Fig. 7a). Consistent with TIM-3 and PD-1 protein expression, T cells isolated from OSM-overexpressing mice presented enrichment of genes associated with T-cell exhaustion^40^ (Fig. 4h, i). Moreover, pathway analyses revealed enrichment of genes involved in inflammation (HALLMARK: inflammatory response; Fig. 4j), cytokine signaling (Reactome: cytokine signaling in the immune system; KEGG: cytokine–cytokine receptor interaction), regulation of leukocyte chemotaxis (KEGG: chemokine signaling pathway; GO: regulation of leukocyte chemotaxis) (supplementary Fig. 7b) and epithelial–mesenchymal transition (HALLMARK EPITHELIAL MESENCHYMAL TRANSITION). In addition, transcripts of various proinflammatory cytokines, including *Csf1*, *Csf2*, or *IL6*, and the chemokines *Ccl2*, *Ccl3*, *Ccl4*, *Ccl6, Cxcl2, and Cxcl10*, which are known to recruit PMN-MDSCs, were upregulated (Fig. 4k).^41^ Genes involved in IL6-JAK-STAT3 signaling (HALLMARK: IL6 JAK STAT3 signaling) were also significantly enriched, while genes related to cell stemness, such as *Tcf7* (logFC −3.4, adj. *p* = 0.0005) or Lef1 (logFC −4.1, adj. *p* = 0.0002) were decreased (HALLMARK: WNT Beta Catenin Signaling; supplementary Fig. 7b). *Tcf7* is expressed at low levels in terminally exhausted T cells and is associated with antitumor immune responses,^42^ suggesting that OSM plays a role in promoting T-cell exhaustion. Taken together, these findings indicate that OSM overexpression in mice induces a reduction in total T-cell numbers, increases T-cell exhaustion markers and expands T-cell-suppressing myeloid cells. These results establish a link between the oncogene-STAT5-OSM axis and immunosuppressive effects in both the T-cell and myeloid cell compartments.

### *Osm* deficiency reduces disease activity in models of leukemia

**Fig. 5:**
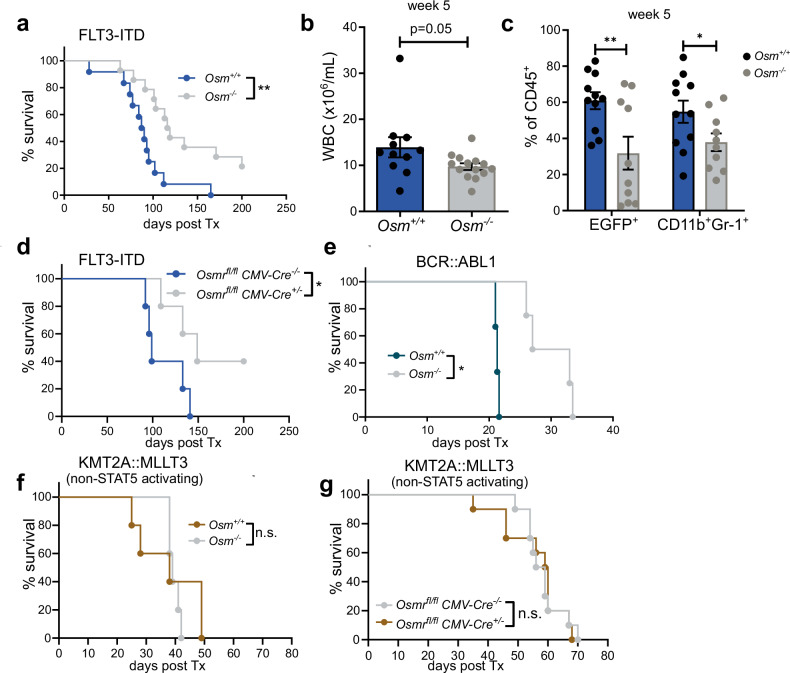
*Osm* deficiency extends survival in models of leukemia with STAT5-activating oncogenes. **a** Kaplan‒Meier plots for survival of recipients transplanted with *Flt3*-ITD^*+*^ bone marrow (BM) from *Osm*^*+/+*^ (WT) vs. *Osm*^*-/-*^ donors. *N* = 12 vs. 14, three independent experiments. **p < 0.01. **b** White blood cell count (WBC) and **c** proportions of CD45^+^EGFP^+^ and CD45^+^CD11b^+^Gr-1^+^ cells five weeks after transplantation in *Flt3*-ITD^+^ mice. *p < 0.05, **p < 0.01. Each dot represents a measurement from one mouse. The data are presented as the means ± SEMs. **d** Kaplan‒Meier plots for survival of *Osmr*^*fl/fl*^ *CMV-Cre*^*-/-*^ vs. *Osmr*^*fl/fl*^ *CMV-Cre*^*+/-*^ recipients transplanted with *Flt3*-ITD^*+*^ BM. *N* = 5 vs. 5. *p < 0.05. **e** Survival with Kaplan‒Meier plots for WT recipients transplanted with *Bcr::Abl1*^*+*^ *Osm*^*+/+*^ vs. *Osm*^*-/-*^ BM. *N* = 3 vs. 4. *p < 0.05. **f**, **g** Kaplan‒Meier plots for **f** recipients transplanted with *Mll-Af9*^*+*^ *Osm*^*+/+*^ vs. *Osm*^*-/-*^ BM or **g** *Osmr*^*fl/fl*^ *CMV-Cre*^*-/-*^ vs. *Osmr*^*fl/fl*^ *CMV-Cre*^*+/-*^ recipients receiving *Mll-Af9*^*+*^ BM. *N* = 5 vs. 5, and 10 vs. 10, respectively

To understand whether OSM is functionally relevant for the disease progression of leukemia, we used *Osm*-deficient mice or WT mice as BM donors in models of FLT3-ITD- and BCR::ABL1-positive leukemia. Compared with *Osm*^*-/*-^ mice, mice receiving FLT3-ITD^+^ *Osm*^*+/+*^ BM had a significantly shorter median survival (Fig. 5a). Additionally, compared with *Osm*^*-/-*^ mice, mice receiving *Flt3*-ITD^+^ *Osm*^*+/+*^ BM had a greater white blood cell (WBC) count and greater expansion of EGFP^+^ and CD11b^+^Gr-1^+^ cells (Fig. 5b, c). *Osmr*^*fl/fl*^ *CMV-Cre*^*+/-*^ mice receiving *Flt3*-ITD^*+*^ BM had longer median survival than *CMV-Cre*^*-/-*^ mice did (Fig. 5d). The survival of the mice transplanted with *Osm*^*+/+*^ BM transduced with *Bcr::Abl1* was lower than that of the *Bcr::Abl1*^*+*^ *Osm*^*-/-*^ BM recipients (Fig. 5e). We also implemented an oncogene-driven leukemia model (KMT2A::MLLT3) in which STAT5 is inactive and OSM is not induced. In addition to the role of OSM in STAT-5-driven leukemia, OSM deficiency in donor BM or the injection of *Kmt2a::Mllt3*^*+*^ BM into *Osmr-*deficient mice had no effect on median survival, WBC count, or lineage distribution compared with that in WT mice (Fig. 5f, g).

### *Osm* deficiency impairs disease induction in a mouse model of MPN

**Fig. 6:**
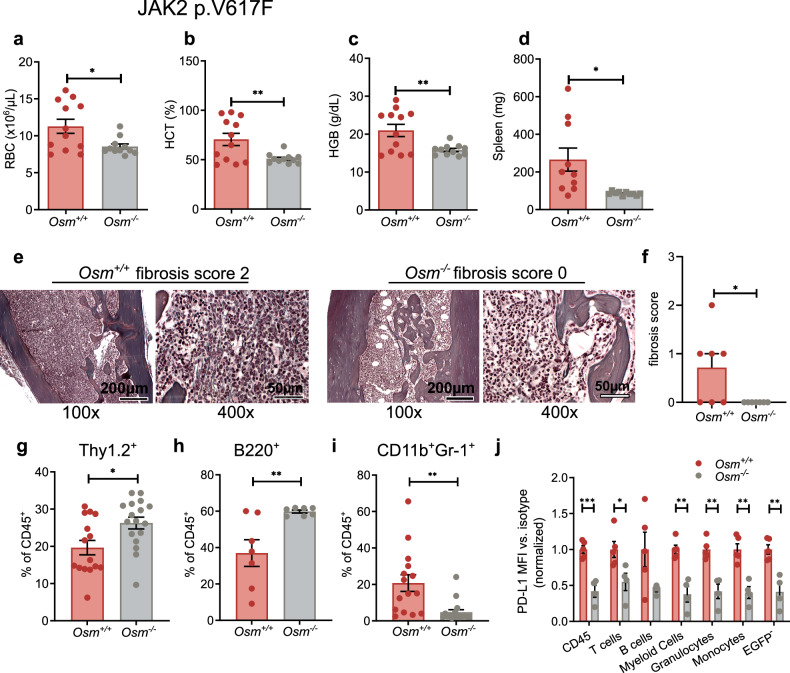
*Osm* deficiency impairs the MPN phenotype in mice transplanted with *Jak2 p.V617F*^*+*^ BM. **a**–**c** WT recipient mice were transplanted with *Jak2 p.V617F*^*+*^ *Osm*^*+/+*^ *or Osm*^*-/-*^ BM. The red blood cell (RBC) count (**a**), hematocrit (HCT) (**b**), and hemoglobin (HGB) (**c**) eight weeks after transplantation are depicted. *N* = 12 vs. 11, *p < 0.05, **p < 0.01. The data are presented as the means ± SEMs. **d** Spleen weights of *Jak2 p.V617F*^*+*^ mice 10 weeks after transplantation. *N* = 10 vs. 10; *p < 0.05. The data are presented as the means ± SEMs. **e** Gomori reticulin staining of bone specimens from *Jak2 p.V617F Osm*^*+/+*^ (left two) and *Osm*^*-/-*^ (right two) mice. **f** Fibrosis scores of *n* = 7 vs. 7 animals. *p < 0.05. The data are presented as the means ± SEMs. **g**–**i** Lineage analysis of CD45^+^ cells in the spleen via flow cytometry. *N* = 15 vs. 15, three experiments. *p < 0.05, **p < 0.01. The data are presented as the means ± SEMs. **j** Flow cytometric analysis of PD-L1 expression on different subsets of bone marrow-derived hematopoietic cells from *Jak2 p.V617F Osm*^*+/+*^ vs. *Osm*^*-/-*^ mice. The mean fluorescence intensity (MFI) of the isotype control was subtracted from the PD-L1 MFI value. *N* = 5 vs. 4. *p < 0.05, **p < 0.01, ***p < 0.001. The data are presented as the means ± SEMs

We then transplanted *Osm*^*-/-*^ or WT BM transduced with *Jak2 p.V617F*, an oncogene that induces STAT5 activation, into C57Bl/6J WT mice. Eight weeks post-transplantation, the mice that received *Jak2 p.V617F*^*+*^ *Osm*^*-/-*^ BM developed no signs of polycythemia vera (PV), with red blood cell (RBC) count, hematocrit (HCT), and hemoglobin (HGB) values within the normal range, whereas the WT mice presented all the signs of disease (Fig. 6a–d). Compared with those in empty vector controls and *Jak2 p.V617F*^*+*^ *Osm*^*-/*-^ recipients, plasma OSM levels were elevated in *Jak2 p.V617F*^*+*^ *Osm*^*+/+*^ mice (supplementary Fig. 8a). *Osm* deficiency also protected against splenomegaly and myelofibrosis induced by JAK2 p.V617F: *Osm*^*-/-*^ mice displayed no signs of fibrosis after more than 200 days, whereas mild to intermediate fibrosis developed in 4/7 *Jak2 p.V617F*^*+*^ *Osm*^*+/+*^ mice (Fig. 6e, f). Thus, OSM knockout in a JAK2 p.V617F-driven mouse model completely reversed the MPN phenotype and affected the development of BM fibrosis. Compared with mice that received the *Jak2 p.V617F*^*+*^ *Osm*^*-/-*^ BM, those that received the *Jak2 p.V617F*^*+*^ *Osm*^*+/+*^ BM had lower T and B lymphocyte counts and more CD11b^+^Gr1^+^Ly6G^+^ myeloid cells (Fig. 6g–i). These alterations could be attributed primarily to cell-extrinsic effects, as mainly EGFP^-^ cells were affected. JAK2 p.V617F was previously shown to induce PD-L1 expression.^2^ *Osm* deficiency reduced the JAK2 p.V617F-induced increase in PD-L1 expression on the surface of various leukocyte populations in WT mice (Fig. 6j). Given the pressing clinical need to address JAK2 p.V617F-positive MPN and prevent BM fibrosis, we opted to focus our further analyses on JAK2 p.V617F-driven disease.

### JAK2 p.V617F OSM wild-type mice display elevated levels of T-cell exhaustion

**Fig. 7:**
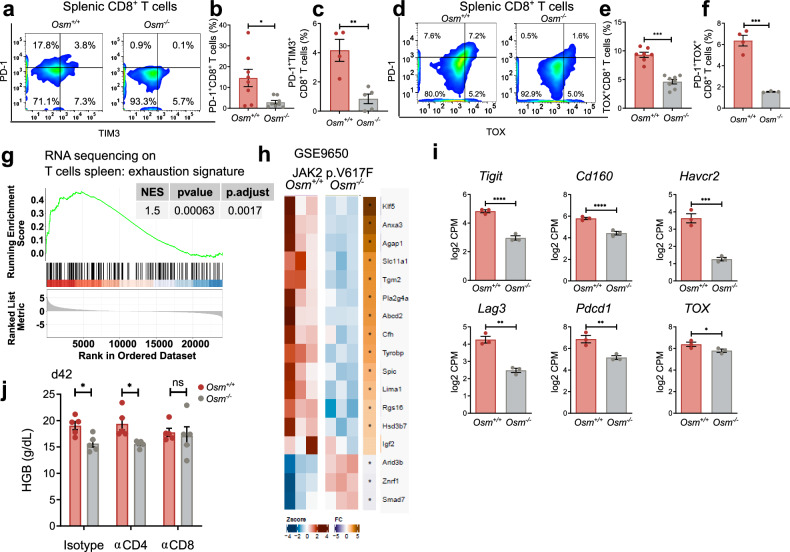
T cells in *Jak2 p.V617F*^*+*^ *Osm*^*+/+*^ BM-transplanted mice display exhaustion. **a**, **d** Flow cytometric analyses of CD8^+^ T cells from the spleens of *Jak2 p.V617F*^*+*^ *Osm*^*+/+*^ vs. *Jak2 p.V617F*^*+*^ *Osm*^*-/-*^ mice for the depicted antigens and **b**, **c**, **e**, **f** statistical analyses of single/double-positive cells. *N* ≥ 3. *p < 0.05, ***p < 0.001. The data are presented as the means ± SEMs. **g** Gene set enrichment analyses of the transcriptome of flow cytometry-sorted bulk CD45^+^Thy1.2^+^ T cells from mice transplanted with *Jak2 p.V617F*^*+*^ *Osm*^*+/+*^ or *Jak2 p.V617F*^*+*^ *Osm*^*-/-*^ BM. Genes upregulated in exhausted samples according to GSE9650^40^ were scored. NES: normalized enrichment score. *N* = 3 for each group. **h** Heatmap displaying exhaustion genes differentially expressed according to GSE9650.^40^ **i** Depiction of specific transcript levels in the BM of mice transplanted with *Jak2 p.V617F*^*+*^ *Osm*^*+/+*^ or *Jak2 p.V617F*^*+*^ *Osm*^*-/-*^. *N* = 3 vs. 3. *p < 0.05, **p < 0.01, ***p < 0.001, ****p < 0.0001. The data are presented as the means ± SEMs. **j** Hemoglobin (HGB) in *Jak2 p.V617F*^*+*^ *Osm*^*+/+*^ vs. *Osm*^*-/-*^ mice treated with CD4- or CD8-depleting antibodies 42 days after BM transplantation. *N* = 5 for each genotype and treatment. *p < 0.05. The data are presented as the means ± SEMs

Consistent with the increase in T-cell exhaustion markers in OSM mice, the expression of the exhaustion markers PD-1, TIM-3, and TOX was greater in CD8^+^ T cells isolated from *Osm*^*+/+*^ mice than in those from *Osm*^*-/-*^ mice (Fig. 7a–f). We measured the levels of reactive oxygen species (ROS), as higher ROS levels were shown to be associated with PMN-MDSC-mediated T-cell suppression.^39^ CellRox staining revealed increased ROS levels in T cells from *JAK2 p.V617F*^*+*^ mice receiving *Osm*^*+/+*^ BM than in those from *Osm*^*-/-*^ BM (supplementary Fig. 8b, c). Transcriptional analysis revealed that T cells isolated from mice that received *Jak2 p.V617F*^*+*^ *Osm*^*+/+*^ BM presented an enrichment of genes indicative of T-cell exhaustion compared with T cells isolated from animals that received *Jak2 p.V617F*^*+*^ *Osm*^*-/-*^ BM (Fig. 7g, h; supplementary Fig. 9a, b). Specifically, genes encoding surface proteins known to be markers of exhaustion, such as *Tigit*, *Havcr2* (encoding TIM-3), *Lag3*, or *Pdcd1*, presented higher transcript levels in T cells from *Jak2 p.V617F*^*+*^ *Osm*^*+/+*^ mice than in T cells isolated from *Jak2 p.V617F*^*+*^ *Osm*^*-/-*^ mice (Fig. 7i). Furthermore, T cells from *Jak2 p.V617F*^*+*^ *Osm*^*+/+*^ mice expressed higher levels of proinflammatory cytokines and chemokines, including *Csf1*, *Csf2*, *IL6*, *Ccl3*, or *Ccl4*, than did T cells isolated from *V617F*^*+*^ *Osm*^*-/-*^ mice (supplementary Fig. 9c). T cells from *Jak2 p.V617F*^*+*^ *Osm*^*+/+*^ mice presented an enrichment of genes associated with IL6-JAK signaling (HALLMARK: IL6 JAK STAT3 signaling; supplementary Fig. 9d).

To understand the role of T cells in the context of OSM/JAK2 p.V617F-driven disease, we used antibody-based T-cell depletion. Transplantation of *Jak2 p.V617F*^*+*^ *Osm*^*+/+*^ or *Osm*^*-/-*^ BM cells into recipient WT mice followed by treatment with antibodies targeting CD4^+^ or CD8^+^ or an isotype control effectively depleted the respective cell population. This finding was confirmed by flow cytometry on days 7, 19, and 42 posttransplantation (supplementary Fig. 10a–c). Isotype-treated *Osm*-deficient mice presented a reduction in the PV-like phenotype with lower HGB levels, which is consistent with our previous observations (Fig. 7j). CD4^+^ T-cell depletion was not sufficient to reverse the PV phenotype (Fig. 7j). However, CD8^+^ T-cell depletion reversed the protective effect of OSM deficiency (Fig. 7j). These results suggest that OSM suppresses CD8^+^ T cells and that this suppression is reversed upon OSM deletion. Furthermore, the PV phenotype re-emerged when CD8^+^ T cells were depleted in the *Jak2 p.V617F Osm*^*-/-*^ disease model.

### Blockade of OSM signaling via a receptor-fusion protein or monoclonal antibody reduces the disease burden in JAK2 p.V617F mice

**Fig. 8:**
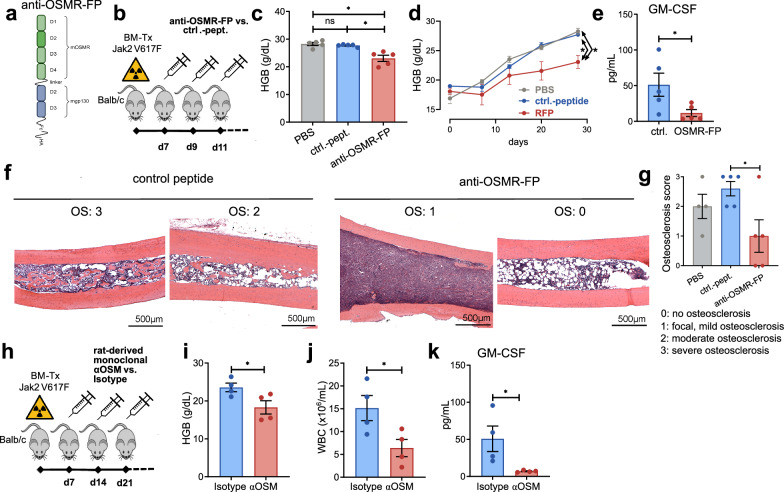
Treatment of JAK2 p.V617F^+^ animals with an anti-OSM receptor fusion protein or monoclonal anti-OSM antibody ameliorates polycythemia and osteosclerosis. **a** Structure of the anti-OSM receptor fusion protein (“anti-OSMR-FP”): the extracellular domains (D) 1–4 of the murine OSMR receptor were fused to D2–3 of murine gp130 and C-terminally Fc- and HA-tagged.^16,43,44^ Figure generated with BioRender. **b** Outline of the in vivo anti-OSMR-FP treatment: Intraperitoneal injections (6 µg/g body weight) were administered every two days from day 7 posttransplantation and maintained for 30 days in *Jak2 p.V617F*^*+*^ BM recipients. *N* = 5 for each treatment group. **c**, **d** Hemoglobin (HGB) in PBS-, control peptide-, and anti-OSMR-FP-treated animals one month after treatment (**c**) and over time (**d**). *N* = 5 for each treatment group. *p < 0.05. The data are presented as the means ± SEMs. **e** Assessment of GM-CSF levels via bead-based flow cytometry in mice 10 weeks after BM transplantation. *p < 0.05. **f** Hematoxylin/eosin staining of bone specimens from JAK2 p.V617F^+^ mice treated with control peptide (left) or anti-OSMR-FP (right). **g** Osteosclerosis score. *N* ≥ 4. *p < 0.05. Statistical analysis was conducted via the Mann‒Whitney test. The data are presented as the means ± SEMs. **h** Outline of the in vivo treatment of mice transplanted with *Jak2 p.V617F*^*+*^ BM with a rat-derived monoclonal anti-mOSM antibody (αOSM). Intraperitoneal injections started on day 7 posttransplantation and were administered weekly for a total of four injections. *N* = 4 for each treatment group. **i**, **j** Hemoglobin (HGB) (**i**) and white blood cell count (WBC) (**j**) in isotype- vs. αOSM-treated animals, as assessed one month after BM transplantation. *N* = 4 vs. 4. *p < 0.05. The data are presented as the means ± SEMs. **k** Evaluation of GM-CSF levels on day 80 posttransplantation via bead-based flow cytometry. *p < 0.05. The data are presented as the means ± SEMs

To evaluate the therapeutic potential of targeting OSM, we utilized a soluble OSM receptor fusion protein (“anti-OSMR-FP”), which binds soluble OSM and prevents its interaction with OSMR (Fig. 8a). The anti-OSMR-FP antibody was produced as previously described,^16,43,44^ and the expected molecular mass was confirmed by immunoblotting (supplementary Fig. 11a). In vitro functional testing demonstrated the ability of anti-OSMR-FP to inhibit mOSM-induced STAT3 phosphorylation in NIH/3T3 cells (supplementary Fig. 11b). The ND_50_ was determined to be 307.5 ng/mL (supplementary Fig. 11b). Additionally, we tested the ND_50_ values for a goat polyclonal anti-mOSM antibody (αmOSM) and a rat monoclonal αmOSM, which were 1091.0 ng/mL and 29.65 ng/mL, respectively (supplementary Fig. 11b). For in vivo experiments, we utilized anti-OSMR-FP and monoclonal rat-derived αmOSM. We administered anti-OSMR-FP, control peptide, or PBS to JAK2 p.V617F^+^ BM-transplanted mice via intraperitoneal (i.p.) injection every second day beginning one week after transplantation (Fig. 8b). After one month of treatment, the HGB and HCT levels were lower in the mice that received anti-OSMR-FP than in those that received PBS or the control peptide (Fig. 8c, d). Furthermore, anti-OSMR-FP-treated mice had lower serum levels of GM-CSF than control peptide-treated mice did (Fig. 8e). In these mice, bone histology revealed a reduction in osteosclerosis (Fig. 8f, g). We subsequently assessed the efficacy of αmOSM in the JAK2 p.V617F model through weekly i.p. injections starting one week after transplantation (Fig. 8h). The schedule was selected on the basis of its efficacy in a murine model of lupus nephritis.^45^ After one month of treatment, the αmOSM-treated mice presented lower HGB, WBC, and GM-CSF levels than the isotype-treated mice did (Fig. 8i–k).

Overall, these results highlight the potential of targeting OSM signaling to mitigate the disease burden in JAK2 p.V617F-driven conditions.

## Discussion

Growing evidence indicates that oncogenic signaling alters the tumor microenvironment to support its own progression.^1–3,46^ We and others have previously shown that OSM is induced by JAK2 p.V617F or FLT3-ITD.^5,7^ We further investigated the effects of OSM on disease progression and fibrosis, its potential for genetic or pharmacologic inhibition, and its effects on T cells and myeloid cells in vivo. We found that in addition to JAK2 p.V617F or FLT3-ITD, the STAT5-activating oncogenes BCR::ABL1 and NPM1::ALK induce OSM. This finding is consistent with the presence of a STAT5 binding site in the promoter region of the OSM gene.^6,47^ *OSM* expression can also be induced by the EP4/cAMP pathway with cyclic AMP response element (CRE)-like sites at the distal Osm promoter site.^48,49^ Inhibition of downstream signaling of oncogenic kinases may be used to interfere with pathogenic OSM production.

We observed that OSMR expression was restricted to the nonhematopoietic BM compartment. Previous studies support this finding: Bisht et al. performed RT‒qPCR on sorted murine BM cells and reported that *Osmr* mRNA was undetectable in BM myeloid cells and HSPCs but was abundantly expressed by endothelial cells and mesenchymal progenitor cells.^16^ Data from the Gene Expression Commons and Hemopedia datasets revealed that *Osmr* is not detected in hematopoietic cells but is abundant in BM CD105^−^ stromal cells and CD45^−^Ter119^−^Tie2^+^ endothelial cells.^28,29^ At the single-cell level (scRNAseq), BM Lepr^+^ mesenchymal cells and Cdh5^+^ endothelial cells were also identified as *Osmr*^+^.^30^ A recent study revealed that the circulating OSMR was the strongest negative predictor in newly diagnosed AML patients.^11^ HY1^−^ MSCs and adipose-derived MSCs presented the highest expression of OSMR, whereas primary AML blasts presented little to no OSMR surface expression (average OSMR^+^ 0.57%). OSMR-high patients presented upregulated IL-6 and OSM expression according to differential protein expression analysis of PB.^11^ In summary, these findings suggest that an important interaction between malignant clones and the MSC niche is mediated by the interaction with OSM/OSMR signaling.

We found that OSM overexpression profoundly reprogrammed BM stromal cells and induced the secretion of the cytokines IL-6 and MCP-1, which are linked to T-cell exhaustion and the induction and expansion of MDSCs.^20,39,50–52^ The connection between OSM and T-cell exhaustion has not been reported in the context of hematological malignancies. However, in mouse models of pancreatic, breast, and hepatocellular cancer, OSM knockout attenuates T-cell exhaustion and enhances immunological tumor control.^19,20^ We overexpressed OSM via the transplantation of retrovirally transduced BM.^5,10^ The transplanted mice presented reduced T-cell numbers, increased levels of T-cell exhaustion markers, and lactic acid (LA) production by stroma cells. Elevated LA levels suppress cytotoxic T-cell proliferation and support regulatory T-cell growth.^37,53^ Furthermore, OSM overexpression led to the expansion of PMN-MDSCs in the spleen. These MDSCs are immunosuppressive in vitro, as they inhibit T-cell proliferation in response to CD3/CD28. MDSCs play a major role in the immunosuppressive TME, as shown for multiple hematological malignancies.^24^ While the role of MDSCs is well established, we demonstrate that OSM induces the expansion of this myeloid cell type and propose OSM depletion as a potential therapeutic strategy to reduce the number of MDSCs. The deletion of *Osm* delayed disease onset in models of myeloid neoplasia driven by the STAT5-activating oncogenes FLT3-ITD or BCR::ABL1. This approach for evaluating the role of OSM in hematological malignancies has not been previously described. In mice with JAK2 p.V617F-driven PV, *Osm* knockout reduced the expression of markers of T-cell exhaustion and disease progression, including polycythemia and myelofibrosis. JAK2 p.V617F-derived neoepitopes are known to be presented by HLA,^54^ suggesting that dysfunctional T-cell immunity may impact the expansion of JAK2 p.V617F^+^ cells. This possibly contributes to the differences observed between *Osm*^+/+^ and *Osm*^*-/-*^ BM-transplanted animals.

As a novel therapeutic intervention, we tested OSM inhibition by a receptor fusion protein and a neutralizing antibody. Both treatments reversed the malignant disease phenotype, including polycythemia, increased levels of proinflammatory cytokines, and osteosclerosis, an effect that is not ameliorated or prevented by ruxolitinib.^55^ Recent research on cytokine-targeted therapies has shown that anti-IL-1β antibody treatment reduces myelofibrosis and osteosclerosis in JAK2 p.V617F mice.^56^ However, anti-IL6 antibody treatment in JAK2 p.V617F knock-in mice resulted in only a modest reduction in cytokine levels and no improvement in polycythemia, leukocytosis, or splenomegaly.^57^ A phase Ib trial of a selective TGFβ 1/3 trap in advanced MF patients showed limited efficacy.^25^ We show that OSM induces the production of numerous cytokines and chemokines from both nonhematopoietic stromal cells and, consequently, hematopoietic cells. This underlines OSM as a master regulator of cytokines, including GM-CSF, IL-1, and IL-6. While targeting IL-6 poses challenges due to potential toxic side effects,^26^ humanized anti-OSM antibodies have been well tolerated in clinical studies in patients with rheumatic disease.^58,59^ Targeting OSM, rather than OSMR, offers the advantage of preferentially blocking highly expressed OSM at sites of active inflammation without affecting OSMR, which is constitutively expressed at various sites.^23^

We next aimed to detect OSM upregulation in primary patient samples. Hoermann et al. reported elevated OSM transcript levels in the BM of patients with MPN and increased OSM serum levels in a subset of MPN patients (15/96), whereas OSM was undetectable in the sera of healthy individuals.^7,8^ In our study, we confirmed elevated OSM transcript levels in PBMCs from MPN patients. Additionally, with our unbiased approach to comprehensively assess OSM levels in AML patients, we observed a clear increase in the number of FLT3-ITD^+^ AML patients with active disease and a trend toward worsened survival in OSM^high^ AML patients. Interestingly, a recent study revealed that circulating MSC-derived OSMR was the strongest negative predictor in newly diagnosed AML patients, whereas OSMR^high^ patients had upregulated IL-6 and OSM according to differential protein expression analysis of PB, as well as enrichment of IL-6/JAK/STAT and the inflammatory response in gene set enrichment analysis.^11^ We therefore propose OSM as a therapeutic target for MPN patients and potentially for those harboring STAT5-activating oncogenes such as FLT3-ITD.

Our study has several limitations. First, while we focused on oncogenes that activate STAT5 and induce OSM (e.g. FLT3-ITD, JAK2 p.V617F, and BCR::ABL1), OSM expression can also be driven by STAT5-independent pathways such as EP4/cAMP signaling.^48,49^ Additionally, although our data demonstrate clear associations between oncogenic STAT5 activation, OSM upregulation, stromal remodeling, and immune dysregulation, many of these findings remain correlative. We did not directly demonstrate that OSM-mediated immune suppression functionally alters tumor cell control in vivo. Future studies should address whether OSM blockade restores antileukemic immunity or synergizes with existing therapies. Checkpoint inhibitors have shown limited success in treating MPNs, often due to immune exhaustion or chronic inflammation.^24,60^ Targeting OSM may offer a distinct approach by disrupting inflammation directly linked to oncogenic signaling.^61^ Unlike general immunosuppressive or checkpoint blockade strategies, OSM inhibition may more specifically reverse disease-promoting inflammation driven by oncogenic pathways. Nevertheless, the cytokine-rich and feedback-driven MPN microenvironment may limit monotherapy effects.^24^ Combining OSM inhibition with other treatments and selecting patients on the basis of OSM/OSMR activity or inflammatory signatures will likely be critical for clinical success. Finally, our work has focused primarily on T cells and MDSCs, whereas the roles of NK cells, dendritic cells, and monocytes remain less explored. Nevertheless, both our group and others have observed predominant effects on T cells and granulocytes in OSM mice as well as in JAK2 p.V617F mice.^10,62^ Moreover, our CD8⁺ T-cell depletion experiments further confirmed the central role of this immune cell subset in the pathophysiology of JAK2 p.V617F.

In summary, our current work elucidates protumorigenic crosstalk involving hematopoietic cancer cells with activated STAT5, which produces OSM. This in turn acts on BM stromal cells expressing OSMR. In this system, OSM induces reprogramming of BM stromal cells, cytokine secretion, and myelofibrosis, accompanied by increased levels of exhaustion markers on T cells and inhibited MDSC recruitment. JAK2 p.V617F-induced PV as well as subsequent myelofibrosis can be successfully reversed by OSM inhibition. Recent reports highlight the involvement of IL-6 family cytokine signaling and chronic inflammation in resistance to immune checkpoint inhibition-based therapies.^52,63^ However, the implications of OSM-OSMR signaling in cancer immunotherapy remain relatively unexplored. Therefore, investigating the potential synergy between OSM/OSMR inhibition and immunotherapy-based approaches may offer promising strategies to improve outcomes for affected patients.

## Materials and methods

### Patient samples and quantification of OSM levels

PB- or BM-derived mononuclear cells from 63 patients (aged 35–83 years) with AML and 13 patients with CML at different stages of disease (primary diagnosis, remission, and relapse for AML or chronic phase, accelerated phase or blast crisis for CML) or 23 healthy donors were analyzed. For *OSMR*-qPCR, mononuclear cells were isolated via a Ficoll density gradient. The cells were stored in liquid nitrogen until further use. A human or murine oncostatin M (OSM) DuoSet ELISA kit (R&D Systems, Minneapolis, MN, USA) was used to quantify the OSM levels in the plasma or cell culture supernatants.

### Transduction and transplantation of murine bone marrow

Our study examined male and female animals, and similar findings have been reported for both sexes. C57BL/6J or BALB/cAn mice aged 6–10 weeks at the time of the experiment were used. *Osm* knockout, *Osmr* conditional knockout, and CMV-Cre mice (all on a C57BL/6J background) were obtained from Jackson Labs (Bar Harbor, Maine, USA). *Osmr*^*fl/fl*^ mice were crossed with mice expressing CMV-Cre recombinase. Retroviral transduction and injection of donor BM cells were performed as previously described.^5^

### Cell culture

32D, NIH/3T3 and OP9 cells were obtained from the German Resource Centre for Biological Material (DSMZ). Phoenix E helper virus-free ecotropic packaging cells were a kind gift from G. Nolan, Stanford, USA. 32D cells were maintained in RPMI 1640 medium (Thermo Fisher, Waltham, MA, USA) containing 10% fetal calf serum (FCS) and 2 ng/mL murine interleukin-3 (IL-3; Peprotech, Hamburg, Germany). Phoenix E, NIH/3T3 and HEK293T cells were maintained in DMEM (Thermo Fisher) supplemented with 10% FCS. OP9 and M2-10B4 cells were cultivated in alpha MEM (Thermo Fisher) supplemented with 10% FCS, EL08-1D2 in alpha MEM supplemented with 15% FCS, 5% horse serum and 50 mM β-mercaptoethanol. MV4-11, MOLM-13, OCI-AML3, HEL, SET-2, and K562 cells were cultured in RPMI 1640 medium supplemented with 10% FCS. Primary human BM stromal cells from healthy BM donors were obtained from our stem cell transplant department and cultured in DMEM supplemented with 10% FCS. All the cells were cultured in 1% penicillin‒streptomycin (Thermo Fisher).

### Cell culture supplements

Recombinant murine and human OMs were obtained from Cell Signaling Technology (Danvers, MA, USA) or PeproTech and were used at a final concentration of 10 ng/mL. Ruxolitinib and sorafenib were purchased from ChemiTek (Indianapolis, IN, USA).

### Retroviral expression vectors and helper virus assay

The retroviral MSCV (murine stem cell virus)-JAK2 p.V617F-internal ribosomal entry site (IRES)-enhanced green fluorescent protein (EGFP) (“MiG-JAK2 p.V617F”), MiG-FLT3-ITD, MiG-BCR::ABL1, and MiG-KMT2A::MLLT3 (supplementary information for sequences) constructs have been described previously.^64^ The construct expressing OSM was a kind gift from J. Schwaller (University Children’s Hospital Basel, Basel, Switzerland).^10^ In this model, murine *Osm* is expressed via a retroviral construct in which the *Osm*-encoding gene is separated from an EGFP cassette by an IRES.^10^ Murine hematopoietic cells from 5-FU-treated wild-type mice were infected with *Osm* retrovirus. As controls, murine hematopoietic cells from 5-FU-treated wild-type mice were transduced with a retrovirus lacking the *Osm* coding sequence.

The high-expressing construct was used in all cases. Transfection of PhoenixE cells and virus harvesting were conducted as previously described.^5^

### Analysis of transplanted mice

Hemoglobin (HGB), hematocrit (HCT), red blood cell (RBC), and platelet counts and white blood cell (WBC) counts were measured via an automated counter (SCIL vet abc, SCIL, Altorf, France). The number of transduced EGFP-positive cells in the PB of the transplanted mice was determined via flow cytometry.

### In vivo T-cell depletion and RFP or anti-mOSM treatment

For in vivo T-cell depletion, the mice received i.p. injections of rat anti-mouse CD4^+^ (clone GK1.5) or CD8a^+^ (clone YTS169.4) antibodies or a rat IgG2b isotype control (LTF-2; all InVivoMAbTM, BioXCell, USA) at a dose of 150 µg/animal starting on day -1 before BM transplantation. For the mOSM antagonizing experiments, the mice were treated with an mOSM-capturing protein (anti-OSM receptor fusion protein, “anti-OSMR-FP”). The production and isolation of anti-OSMR-FP and control peptides were described previously.^16,43,44^ Stably transfected HEK293 producer cells (HEK293 Flp-In T-Rex OSM-RFP-FC-5xV5-5xHA and HEK293 Flp-In T-Rex FC-5xV5-5xHA) were cultured and selected with hygromycin and blasticidin. Protein secretion was induced by doxycycline (100 ng/mL). The supernatant was collected, and the protein was isolated via automated liquid chromatography (ÄKTAprime; Cytiva, Marlborough, MA, USA). The protein concentration was determined via a bicinchoninic acid assay. Anti-OSMR-FP-treated mice received i.p. injections every two days starting one week after transplantation for a period of 30 days at a dose of 6 µg/g body weight. Another cohort of mice received treatment with a rat-derived monoclonal anti-mOSM antibody (MAB4951; R&D Systems, Minneapolis, MN, USA) at a dose of 1 µg/g body weight or a corresponding rat IgG_2B_ (clone 823157, R&D) starting one week after transplantation by weekly i.p. injection.^45^

To assess the ability of RFP or the monoclonal anti-mOSM antibody to block mOSM signaling, NIH/3T3 cells were cocultured with 5 ng/mL mOSM plus the respective inhibitor at decreasing concentrations, and pSTAT3 levels were measured via flow cytometry.

### Histopathology

BM samples were fixed in 4% buffered formalin (pH 7.4), decalcified in EDTA, and subsequently stained with Hematoxylin and Eosin (H&E) and Gomori’s reticulin. Slides were examined using a Zeiss Axioplan 2 microscope equipped with a 40x/0.75NA Plan-Neofluar air objective. Images were captured with a Zeiss Axiocam MRc 5 camera and processed using Axiovision Rel 4.6 software.

### Flow cytometry-based analysis and cell sorting

Flow cytometry analysis was performed on a BD LSR Fortessa™, Beckman CyAn™ ADP, or Sony SP6800 Spectral Analyzer. The cells were sorted on a BD FACSAria III. The antibodies used to stain the following cell surface markers were used: anti-mouse CD11b (Mac-1, M1/70), Gr-1 (RB6-8C5), Ly6C (HK1.4), Ly6G (1A8-Ly6G), CD25 (PC61.5), PD-L1 (10 F.9G2), CD45R/B220 (RA3-6B2), CD90.2 (Thy1.2, 30-H12), CD4 (RM4-5), CD8 (53-6.7), CD45 (30-F11), CD117 (c-kit, 2B8), CD127 (Il-7Ra, A7R34), Sca-1 (D7), CD279 (PD-1, RMP1-30), and CD366 (TIM3, 8B.2C12), all obtained from BD Biosciences or Thermo Fisher.

For sorting, lineage-positive cells were removed via the Lineage Cell Depletion Kit (Miltenyi Biotec). Sorting of murine stroma cell populations, spleens, or BM cells was performed on a BD FACS AriaIII™ as described previously.^65^

For intracellular staining, the cells were treated with Cytofix Buffer and Perm Buffer III (both BD Biosciences) according to the manufacturer’s protocol. A FOXP3/transcription factor kit (eBioscience^TM^, Invitrogen) was used for intracellular staining of FOXP3 or TOX. Staining was performed using pSTAT3 (pY705, clone 49/p-Stat3, BD), TOX (TXRX10; Invitrogen), and FOXP3 (FJK-16s; Invitrogen) antibodies. FlowJo X 10.8.1 analysis software was used to analyze the flow cytometry data.

### Coculture experiments

For the T-cell/MDSC coculture experiments, T cells were isolated via the “Pan T-Cell Isolation Kit II, mouse” (magnetic-activated cell sorting, MACS Miltenyi Biotec, Bergisch Gladbach, Germany) and activated with the mouse T-Activator CD3/CD28 Dynabeads^TM^ (Thermo Fisher Scientific). Myeloid-derived suppressor cells (MDSCs) were isolated via the “Myeloid-Derived-Suppressor Cell Isolation Kit, mouse” (MACS Miltenyi Biotec). The cells were cultured at a density of 2 × 10^5^ cells/well in a 96-well flat-bottom plate. Cell proliferation was assessed via the Click-iT™ EdU Alexa Fluor™ 647 Flow Cytometry Assay Kit (Thermo Fisher) according to the manufacturer’s instructions. EdU was added 1 day after coculture, and fixation, surface staining, and flow cytometry (triplicates) were performed the following day.

### Cytokine array

Mouse blood was collected in serum collection tubes (Microtainer; BD Bioscience) and allowed to clot at room temperature for 30 min, followed by centrifugation (1000 × *g*, RT, 10 min) to isolate serum. Cytokine levels in the serum were measured via the LEGENDplex™ Mouse Inflammation Panel (13-plex) (BioLegend) according to the manufacturer’s instructions. Serum samples were analyzed in duplicate (25 µL each).

### Quantitative RT‒PCR analysis

RNA was extracted via the RNeasy Plus Mini Kit (QIAGEN, Hilden, Germany) and transcribed into cDNA via the RevertAid First Strand cDNA Synthesis Kit (Thermo Scientific) according to the manufacturers’ protocol. Quantitative RT‒PCR was performed using a LightCycler™ instrument II (Roche) and LightCycler® 480 SYBR Green I Master Mix (Roche). The sequences of primers used for quantitative RT‒PCR were as follows:

**Table:** 

muOSM for	5’ tgagaggaagagttggagca 3’
muOSM rev	5’ aacactgctcagtttgaccct 3’
mGAPDH for	5’ ttcaccaccatggagaaggc 3’
mGAPDH rev	5’ ggcatggactgtggtcatga 3’
huIL6 for	5’ agacagccactcacctcttcag 3’
huIL6 rev	5’ ttctgccagtgcctctttgctg 3’
huCCL2 for	5’ agaatcaccagcagcaagtgtcc 3’
huCCL2 rev	5’ tcctgaacccacttctgcttgg 3’
hCSF2 for	5’ ggagcatgtgaatgccatccag 3’
hCSF2 rev	5’ ctggaggtcaaacatttctgagat 3’
huGAPDH for	5’ gtcagtggtggacctgacct3’
huGAPDH rev	5’ tgagcttgacaaagtggtcg 3’

### Metabolic rates

The oxygen consumption rate (OCR) and the extracellular acidification rate (ECAR) were measured via an Agilent Seahorse XFe 96 Analyzer (Agilent, Santa Clara, CA, USA). The optimal cell numbers and compound concentrations were determined experimentally according to the manufacturer’s recommendations. Twenty-four hours prior to the assay, the cells were plated on XFe96/XF Pro Cell Culture Microplates (Agilent, CA, USA) at a density of 7–15 × 10^3^ cells/well (4 replicates) with or without mOSM in the appropriate culture medium. On the day of the experiment, the cells were washed and incubated in XF base medium supplemented with 2 mM L-glutamine, pH = 7.4, for 1 h in a non-CO_2_ incubator. For the OCR analysis, the base medium additionally contained 1 mM sodium pyruvate and 25 mM D-glucose (all from Sigma-Aldrich, Steinheim, Germany). Oligomycin A (1 µM), carbonyl cyanide p-trifluoromethoxyphenylhydrazone (FCCP, 1.5 µM), and rotenone (100 nM) plus antimycin A (1 µM; all Sigma-Aldrich, Steinheim, Germany) were sequentially injected after loading into XFe96/XF Pro sensor cartridges to measure basal respiration, ATP production, maximal respiration, and spare capacity, respectively. For the ECAR measurements, D-glucose (10 mM), oligomycin A (1 µM), and 2-deoxy-glucose (50 mM, Sigma-Aldrich, Steinheim, Germany) were injected. The results were analyzed via Seahorse XFe96 Controller Software v2.4.3.7.

### Metabolomics

For low-input targeted metabolomics of (semi)polar metabolites, 500,000 cells were washed with 1000 μL of 2.8% glycerol solution, and metabolites were extracted with 100 μL of 80% methanol containing 13 C yeast extract as an internal standard. Samples were vacuum concentrated using an EZ2 elite (Genevac) and stored at −80 °C until further processing. Targeted metabolite quantification was performed by LC‒MS using an Agilent 1290 Infinity II UHPLC coupled to an Agilent 6495 QQQ‒MS operating in MRM mode. MRM parameters were individually optimized for each compounds using pure standards. LC separation was achieved on a Phenomenex Luna propylamine column (50 × 2 mm, 3 mm particles) with a gradient from 100% buffer B (5 mM ammonium carbonate in 90% acetonitrile) to 90% buffer A (10 mM NH4 in water), and the flow rate was adjusted from 1000 to 750 μL/min. The autosampler was maintained at 5 °C, and the injection volume was 2 μL. Data were processed using Agilent MassHunter software (Version B.08.00), and metabolite abundance was quantified as the area under the curve (AUC). Each data point represents a biological replicate.

### Reactive oxygen species assay

To measure the levels of reactive oxygen species (ROS) in cells, we utilized the CellROX Flow Cytometry Kit (Thermo Fisher). Briefly, the cells were treated with 5 µM CellROX Deep Red reagent (or DMSO) for 30 min at 37 °C. The cells were subsequently washed, surface stained, and analyzed via flow cytometry. As a negative control, cells were preincubated with 50 µM N-acetylcysteine (NAC; Thermo Fisher) before CellROX treatment.

### Transcriptomic analysis

Transcriptome analysis was conducted on RNA isolated from flow cytometry-sorted murine splenic bulk splenocytes (CD45^+^Thy1.2^+^). RNA quality was assessed via an Agilent 4200 TapeStation (Agilent Technologies, Santa Clara, CA, USA). Sequencing was performed with an Ultra Low Input RNA library preparation kit and sequenced on an Illumina HiSeq 4000 at the DKFZ core facility in Heidelberg, Germany. The paired-end reads were trimmed to eliminate adapter sequences and poor-quality reads. Trimmomatic (v0.38)^66^ was applied with the following parameters: HEADCROP:3 TRAILING:10 MINLEN:25. Gene read counts were then determined through the STAR^67^ aligner (v2.5.2b) using the following settings: --outFilterMultimapNmax 10 --alignSJoverhangMin 8 --alignSJDBoverhangMin 1 --outFilterMismatchNmax 999 --outFilterMismatchNoverLmax 0.05 --alignIntronMin 20 --alignIntronMax 1000000 --alignMatesGapMax 1000000 --outFilterMatchNmin 16. The STAR index was built via the mm10 genome from Ensembl.

Subsequent analysis was conducted via the R (v4.2.2) and Bioconductor packages. Differential gene expression analysis was performed via the linear-model-based limma (v3.54.2) R package.^68^ The enriched gene sets were determined through the clusterProfiler (v4.6.2) R package^69^ via the MSigDB reference gene sets.^70^ An adjusted *p* value less than 0.05 was considered significant in both analyses.

### Immunoblot

Immunoblotting of the HEK293T cell supernatant was performed as previously described.^64,71^ Antibodies against human influenza hemagglutinin (HA) were purchased from Cell Signaling. Bands were visualized via an enhanced chemiluminescence (ECL) system (Amersham, Braunschweig, Germany).

### *OSM* and *OSMR* expression analysis via the DepMap portal

The expression of *OSM* and *OSMR* was investigated via the DepMap portal (https://depmap.org) for each gene, utilizing Expression Public 24Q2 and grouping for lineage subtypes.

### Statistical analysis

*P* values were calculated via two-sided Student’s t tests via Prism software (GraphPad, La Jolla, CA, USA), unless otherwise specified in the figure legend. The data represent the means ± SEMs. Sample sizes were estimated in cooperation with the Center for Medical Biometry and Medical Informatics at the University of Freiburg.

### Study approval

This study was conducted in accordance with the Declaration of Helsinki. All healthy controls and patients provided written informed consent. The study was approved by the Ethics Committee of Freiburg University Medical Center (protocol numbers 182/20 and 292/20).

The mice were housed in a specialized caging system with autoclaved food and acidified water at the University Hospital Freiburg, adhering to national and institutional guidelines for animal care. All animal procedures were approved by the regional review board (registration nos. G18/128 and G24/003).

## Supplementary information

Supplementary Data

## Data Availability

The gene expression profiles described in this study can be found at the Gene Expression Omnibus, which is available online. Accession numbers: GSE280988 and GSE280685.

## References

[1] Mathew, N. R. et al. Sorafenib promotes graft-versus-leukemia activity in mice and humans through IL-15 production in FLT3-ITD mutant leukemia cells. *Nat. Med.* **24**, 282–291 (2018). 29431743 10.1038/nm.4484 PMC6029618

[2] Prestipino, A. et al. Oncogenic JAK2V617F causes PD-L1 expression, mediating immune escape in myeloproliferative neoplasms. *Sci. Transl. Med.* **10**, eaam7729 (2018). 29467301 10.1126/scitranslmed.aam7729 PMC6034655

[3] Schmidt, D. et al. Oncogenic calreticulin induces immune escape by stimulating TGF-á expression and regulatory T cell expansion in the bone marrow microenvironment. *Cancer Res.* **84**, 2985–3003 (2024). 38885318 10.1158/0008-5472.CAN-23-3553 PMC11405138

[4] Tefferi, A. & Barbui, T. Polycythemia vera and essential thrombocythemia: 2021 update on diagnosis, risk-stratification and management. *Am. J. Hematol.* **95**, 1599–1613 (2020). 32974939 10.1002/ajh.26008

[5] Müller, T. A. et al. Lineage-specific STAT5 target gene activation in hematopoietic progenitor cells predicts the FLT3(+)-mediated leukemic phenotype. *Leukemia* **30**, 1725–1733 (2016). 27046463 10.1038/leu.2016.72

[6] Yoshimura, A. et al. Mouse oncostatin M: an immediate early gene induced by multiple cytokines through the JAK-STAT5 pathway. *EMBO J.* **15**, 1055–1063 (1996). 8605875 PMC450003

[7] Hoermann, G. et al. Identification of oncostatin M as a JAK2 V617F-dependent amplifier of cytokine production and bone marrow remodeling in myeloproliferative neoplasms. *FASEB J.* **26**, 894–906 (2012). 22051730 10.1096/fj.11-193078

[8] Hoermann, G. et al. Identification of oncostatin M as a STAT5-dependent mediator of bone marrow remodeling in KIT D816V-positive systemic mastocytosis. *Am. J. Pathol.* **178**, 2344–2356 (2011). 21457934 10.1016/j.ajpath.2011.01.020 PMC3081146

[9] Hoermann, G. et al. Oncostatin M is a FIP1L1/PDGFRA-dependent mediator of cytokine production in chronic eosinophilic leukemia. *Allergy* **68**, 713–723 (2013). 23621172 10.1111/all.12139

[10] Schwaller, J. et al. Stat5 is essential for the myelo- and lymphoproliferative disease induced by TEL/JAK2. *Mol. Cell* **6**, 693–704 (2000). 11030348 10.1016/s1097-2765(00)00067-8

[11] Reville, P. K. et al. Blood-based proteomic profiling identifies OSMR as a novel biomarker of AML outcomes. *Blood* **145**, 3015–3029 (2025). 40179376 10.1182/blood.2024027244 PMC12226760

[12] Ichihara, M., Hara, T., Kim, H., Murate, T. & Miyajima, A. Oncostatin M and leukemia inhibitory factor do not use the same functional receptor in mice. *Blood* **90**, 165–173 (1997). 9207450

[13] Boulanger, M. J., Bankovich, A. J., Kortemme, T., Baker, D. & Garcia, K. C. Convergent mechanisms for recognition of divergent cytokines by the shared signaling receptor gp130. *Mol. Cell* **12**, 577–589 (2003). 14527405 10.1016/s1097-2765(03)00365-4

[14] Lindberg, R. A. et al. Cloning and characterization of a specific receptor for mouse oncostatin M. *Mol. Cell. Biol.* **18**, 3357–3367 (1998). 9584176 10.1128/mcb.18.6.3357 PMC108917

[15] West, N. R. et al. Oncostatin M drives intestinal inflammation and predicts response to tumor necrosis factor-neutralizing therapy in patients with inflammatory bowel disease. *Nat. Med.* **23**, 579–589 (2017). 28368383 10.1038/nm.4307 PMC5420447

[16] Bisht, K. et al. Oncostatin M regulates hematopoietic stem cell (HSC) niches in the bone marrow to restrict HSC mobilization. *Leukemia* **36**, 333–347 (2022). 34518644 10.1038/s41375-021-01413-z

[17] Schnittker, D., Kwofie, K., Ashkar, A., Trigatti, B. & Richards, C. D. Oncostatin M and TLR-4 ligand synergize to induce MCP-1, IL-6, and VEGF in human aortic adventitial fibroblasts and smooth muscle cells. *Mediators Inflamm.* **2013**, 317503 (2013). 24307759 10.1155/2013/317503 PMC3836373

[18] Richards, C. D., Langdon, C., Deschamps, P., Pennica, D. & Shaughnessy, S. G. Stimulation of osteoclast differentiation in vitro by mouse oncostatin M, leukaemia inhibitory factor, cardiotrophin-1 and interleukin 6: synergy with dexamethasone. *Cytokine* **12**, 613–621 (2000). 10843736 10.1006/cyto.1999.0635

[19] Araujo, A. M. et al. Stromal oncostatin M cytokine promotes breast cancer progression by reprogramming the tumor microenvironment. *J. Clin. Invest.* **132**, e148667 (2022). 35192545 10.1172/JCI148667 PMC8970678

[20] Lee, B. Y. et al. Heterocellular OSM-OSMR signalling reprograms fibroblasts to promote pancreatic cancer growth and metastasis. *Nat. Commun.* **12**, 7336 (2021). 34921158 10.1038/s41467-021-27607-8 PMC8683436

[21] Langdon, C., Leith, J., Smith, F. & Richards, C. D. Oncostatin M stimulates monocyte chemoattractant protein-1- and interleukin-1-induced matrix metalloproteinase-1 production by human synovial fibroblasts in vitro. *Arthritis Rheum.* **40**, 2139–2146 (1997). 9416850 10.1002/art.1780401207

[22] Minehata, K. et al. Oncostatin m maintains the hematopoietic microenvironment and retains hematopoietic progenitors in the bone marrow. *Int. J. Hematol.* **84**, 319–327 (2006). 17118758 10.1532/IJH97.06090

[23] West, N. R., Owens, B. M. J. & Hegazy, A. N. The oncostatin M-stromal cell axis in health and disease. *Scand. J. Immunol.* **88**, e12694 (2018). 29926972 10.1111/sji.12694

[24] Braun, L. M. & Zeiser, R. Immunotherapy in Myeloproliferative Diseases. *Cells* **9**, 1559 (2020). 32604862 10.3390/cells9061559 PMC7349594

[25] Mascarenhas, J. et al. A Phase Ib Trial of AVID200, a TGFβ 1/3 Trap, in Patients with Myelofibrosis. *Clin. Cancer Res.* **29**, 3622–3632 (2023). 37439808 10.1158/1078-0432.CCR-23-0276 PMC10502472

[26] Kang, S., Tanaka, T., Narazaki, M. & Kishimoto, T. Targeting interleukin-6 signaling in clinic. *Immunity* **50**, 1007–1023 (2019). 30995492 10.1016/j.immuni.2019.03.026

[27] Junk, D. J. et al. Oncostatin M promotes cancer cell plasticity through cooperative STAT3-SMAD3 signaling. *Oncogene* **36**, 4001–4013 (2017). 28288136 10.1038/onc.2017.33 PMC5509502

[28] Seita, J. et al. Gene expression commons: an open platform for absolute gene expression profiling. *PLoS ONE* **7**, e40321 (2012). 22815738 10.1371/journal.pone.0040321 PMC3399844

[29] Choi, J. et al. Haemopedia RNA-seq: a database of gene expression during haematopoiesis in mice and humans. *Nucleic Acids Res.* **47**, D780–D785 (2019). 30395284 10.1093/nar/gky1020 PMC6324085

[30] Tikhonova, A. N. et al. The bone marrow microenvironment at single-cell resolution. *Nature* **569**, 222–228 (2019). 30971824 10.1038/s41586-019-1104-8 PMC6607432

[31] Haferlach, T. et al. Clinical utility of microarray-based gene expression profiling in the diagnosis and subclassification of leukemia: report from the International Microarray Innovations in Leukemia Study Group. *J. Clin. Oncol.* **28**, 2529–2537 (2010). 20406941 10.1200/JCO.2009.23.4732 PMC5569671

[32] Tyner, J. W. et al. Functional genomic landscape of acute myeloid leukaemia. *Nature* **562**, 526–531 (2018). 30333627 10.1038/s41586-018-0623-z PMC6280667

[33] Ogawa, S., Satake, M. & Ikuta, K. Physical and functional interactions between STAT5 and Runx transcription factors. *J. Biochem.* **143**, 695–709 (2008). 18296717 10.1093/jb/mvn022

[34] Tanaka, M. et al. Targeted disruption of oncostatin M receptor results in altered hematopoiesis. *Blood* **102**, 3154–3162 (2003). 12855584 10.1182/blood-2003-02-0367

[35] Stephens, J. M. & Elks, C. M. Oncostatin M: potential implications for malignancy and metabolism. *Curr. Pharm. Des.* **23**, 3645–3657 (2017). 28677505 10.2174/1381612823666170704122559

[36] Oostendorp, R. A. J. et al. Long-term maintenance of hematopoietic stem cells does not require contact with embryo-derived stromal cells in cocultures. *Stem Cells* **23**, 842–851 (2005). 15917480 10.1634/stemcells.2004-0120

[37] Uhl, F. M. et al. Metabolic reprogramming of donor T cells enhances graft-versus-leukemia effects in mice and humans. *Sci. Transl. Med.* **12**, eabb8969 (2020). 33115954 10.1126/scitranslmed.abb8969 PMC8529950

[38] Han, L. et al. Multifaceted oncostatin M: novel roles and therapeutic potential of the oncostatin M signaling in rheumatoid arthritis. *Front. Immunol.* **14**, 1258765 (2023). 38022540 10.3389/fimmu.2023.1258765 PMC10654622

[39] Gabrilovich, D. I. Myeloid-derived suppressor cells. *Cancer Immunol. Res.* **5**, 3–8 (2017). 28052991 10.1158/2326-6066.CIR-16-0297 PMC5426480

[40] Wherry, E. J. et al. Molecular signature of CD8+ T cell exhaustion during chronic viral infection. *Immunity* **27**, 670–684 (2007). 17950003 10.1016/j.immuni.2007.09.006

[41] Owen, J. L. et al. Expression of the inflammatory chemokines CCL2, CCL5 and CXCL2 and the receptors CCR1-3 and CXCR2 in T lymphocytes from mammary tumor-bearing mice. *Cell. Immunol.* **270**, 172–182 (2011). 21621198 10.1016/j.cellimm.2011.05.004 PMC3156845

[42] Talvard-Balland, N. et al. Oncogene induced TIM-3 ligand expression dictates susceptibility to anti-TIM-3 therapy in mice. *J. Clin. Invest.* **134**, e177460 (2024). 38916965 10.1172/JCI177460 PMC11324309

[43] Brolund, L., Küster, A., Korr, S., Vogt, M. & Müller-Newen, G. A receptor fusion protein for the inhibition of murine oncostatin M. *BMC Biotechnol.* **11**, 3 (2011). 21223559 10.1186/1472-6750-11-3 PMC3040522

[44] Schwache, D. & Müller-Newen, G. Receptor fusion proteins for the inhibition of cytokines. *Eur. J. Cell Biol.* **91**, 428–434 (2012). 21958554 10.1016/j.ejcb.2011.07.008

[45] Liu, Q. et al. Anti-OSM antibody inhibits tubulointerstitial lesion in a murine model of lupus nephritis. *Mediators Inflamm.* **2017**, 3038514 (2017). 28626343 10.1155/2017/3038514 PMC5463100

[46] Rakhra, K. et al. CD4(+) T cells contribute to the remodeling of the microenvironment required for sustained tumor regression upon oncogene inactivation. *Cancer Cell* **18**, 485–498 (2010). 21035406 10.1016/j.ccr.2010.10.002 PMC2991103

[47] Yongsheng, M., Raphael, J. S., Jingwen, L., Michael, J. S. & Vestal, R. E. Cloning and characterization of human oncostatin M promoter. *Nucleic Acids Res.* **27**, 4649–4657 (1999). 10556323 10.1093/nar/27.23.4649 PMC148755

[48] Mukherjee, S. & Sengupta Bandyopadhyay, S. Mechanism of prostaglandin E2-induced transcriptional up-regulation of Oncostatin-M by CREB and Sp1. *Biochem. J.* **475**, 477–494 (2018). 29269396 10.1042/BCJ20170545

[49] Ganesh, K. et al. Prostaglandin E₂ induces oncostatin M expression in human chronic wound macrophages through Axl receptor tyrosine kinase pathway. *J. Immunol.* **189**, 2563–2573 (2012). 22844123 10.4049/jimmunol.1102762 PMC3438225

[50] Kato, T. et al. Correlations of programmed death 1 expression and serum IL-6 level with exhaustion of cytomegalovirus-specific T cells after allogeneic hematopoietic stem cell transplantation. *Cell. Immunol.* **288**, 53–59 (2014). 24657340 10.1016/j.cellimm.2014.02.007

[51] Weber, R. et al. IL-6 as a major regulator of MDSC activity and possible target for cancer immunotherapy. *Cell. Immunol.* **359**, 104254 (2021). 33296753 10.1016/j.cellimm.2020.104254

[52] Soler, M. F., Abaurrea, A., Azcoaga, P., Araujo, A. M. & Caffarel, M. M. New perspectives in cancer immunotherapy: targeting IL-6 cytokine family. *J. Immunother. Cancer* **11**, e007530 (2023). 37945321 10.1136/jitc-2023-007530 PMC10649711

[53] Watson, M. J. et al. Metabolic support of tumour-infiltrating regulatory T cells by lactic acid. *Nature* **591**, 645–651 (2021). 33589820 10.1038/s41586-020-03045-2 PMC7990682

[54] Ivanova, M. et al. Probable HLA-mediated immunoediting of JAK2 V617F-driven oncogenesis. *Exp. Hematol.* **92**, 75–88.e10 (2020). 33017633 10.1016/j.exphem.2020.09.200

[55] Verstovsek, S. et al. Safety and efficacy of INCB018424, a JAK1 and JAK2 inhibitor, in myelofibrosis. *N. Engl. J. Med.* **363**, 1117–1127 (2010). 20843246 10.1056/NEJMoa1002028 PMC5187954

[56] Rai, S. et al. Inhibition of interleukin-1β reduces myelofibrosis and osteosclerosis in mice with JAK2-V617F driven myeloproliferative neoplasm. *Nat. Commun.* **13**, 5346 (2022). 36100613 10.1038/s41467-022-32927-4 PMC9470591

[57] Baldauf, C. K. et al. Anti-IL-6 cytokine treatment has no impact on elevated hematocrit and splenomegaly in a polycythemia vera mouse model. *Blood Adv.* **6**, 399–404 (2022). 34559181 10.1182/bloodadvances.2021004379 PMC8791576

[58] Reid, J. et al. In vivo affinity and target engagement in skin and blood in a first-time-in-human study of an anti-oncostatin M monoclonal antibody. *Br. J. Clin. Pharmacol.* **84**, 2280–2291 (2018). 29900565 10.1111/bcp.13669 PMC6138480

[59] Choy, E. H. et al. Safety, tolerability, pharmacokinetics and pharmacodynamics of an anti-oncostatin M monoclonal antibody in rheumatoid arthritis: results from phase II randomized, placebo-controlled trials. *Arthritis Res. Ther.* **15**, R132 (2013). 24286335 10.1186/ar4312 PMC3978888

[60] Hobbs, G. et al. PD-1 inhibition in advanced myeloproliferative neoplasms. *Blood Adv.* **5**, 5086–5097 (2021). 34581778 10.1182/bloodadvances.2021005491 PMC9152999

[61] Gorantla, S. P. et al. Efficacy of JAK1/2 inhibition in murine myeloproliferative neoplasms is not mediated by targeting oncogenic signaling. *Nat. Commun.* **16**, 4833 (2025). 40413183 10.1038/s41467-025-60019-6 PMC12103521

[62] Juan, T. S.-C. et al. Mice overexpressing murine oncostatin M (OSM) exhibit changes in hematopoietic and other organs that are distinct from those of mice overexpressing human OSM or bovine OSM. *Vet. Pathol.* **46**, 124–137 (2009). 19112126 10.1354/vp.46-1-124

[63] Huseni, M. A. et al. CD8+ T cell-intrinsic IL-6 signaling promotes resistance to anti-PD-L1 immunotherapy. *Cell Rep. Med.* **4**, 100878 (2023). 36599350 10.1016/j.xcrm.2022.100878 PMC9873827

[64] Gorantla, S. P. et al. Oncogenic JAK2V617F requires an intact SH2-like domain for constitutive activation and induction of a myeloproliferative disease in mice. *Blood* **116**, 4600–4611 (2010). 20696946 10.1182/blood-2009-07-236133

[65] Müller, T. A. et al. PIM1 inhibition effectively enhances plerixafor-induced HSC mobilization by counteracting CXCR4 upregulation and blocking CXCL12 secretion. *Leukemia* **33**, 1296–1301 (2019). 30816332 10.1038/s41375-019-0428-6

[66] Bolger, A. M., Lohse, M. & Usadel, B. Trimmomatic: a flexible trimmer for Illumina sequence data. *Bioinformatics* **30**, 2114–2120 (2014). 24695404 10.1093/bioinformatics/btu170 PMC4103590

[67] Dobin, A. et al. STAR: ultrafast universal RNA-seq aligner. *Bioinformatics* **29**, 15–21 (2013). 23104886 10.1093/bioinformatics/bts635 PMC3530905

[68] Ritchie, M. E. et al. limma powers differential expression analyses for RNA-sequencing and microarray studies. *Nucleic Acids Res.* **43**, e47 (2015). 25605792 10.1093/nar/gkv007 PMC4402510

[69] Wu, T. et al. clusterProfiler 4.0: a universal enrichment tool for interpreting omics data. *Innovation* **2**, 100141 (2021). 34557778 10.1016/j.xinn.2021.100141 PMC8454663

[70] Liberzon, A. et al. The Molecular Signatures Database (MSigDB) hallmark gene set collection. *Cell Syst.* **1**, 417–425 (2015). 26771021 10.1016/j.cels.2015.12.004 PMC4707969

[71] Jakob, L. et al. Murine Oncostatin M has opposing effects on the proliferation of OP9 bone marrow stromal cells and NIH/3T3 fibroblasts signaling through the OSMR. *Int. J. Mol. Sci.* **22**, 11649 (2021). 34769079 10.3390/ijms222111649 PMC8584221

