# Supplementary material for: Oncostatin M induced by STAT5-activating oncogenes promotes disease progression in hematologic malignancies
Source: Signal Transduct Target Ther. 2025 Dec 11;10:400. doi: 10.1038/s41392-025-02491-6 (PMC12696130; doi:10.1038/s41392-025-02491-6)

Supplementary Materials for

**Oncostatin M induced by STAT5-activating oncogenes promotes disease progression in hematologic malignancies**

Michael Rassner, Tony Andreas Müller, Kirstyn Anne Crossley, Geoffroy Andrieux, Sabina Schaberg, Cornelia Endres, Lena Jakob, Teresa Poggio, Natalie Köhler, Julia Kolter, Gerhard Müller-Newen, Katharina Schönberger, Nina Cabezas-Wallscheid, Irene Gonzalez-Menendez, Leticia Quintanilla-Martinez, Melissa Zwick, Driti Ashok, Tanja Nicole Hartmann, Olaf Groß, Oliver Gorka, Marie Follo, Anna Lena Illert, Melanie Boerries, Robert Zeiser, and Justus Duyster

Correspondence to: justus.duyster@uniklinik-freiburg.de

**This PDF file includes:**

Figures S1 to S11

Tables S1 to S2

Supplementary Text

Data S1 (uncropped version of immunoblot depicted in supplementary Fig. S11a)

**Figure S1.**

**
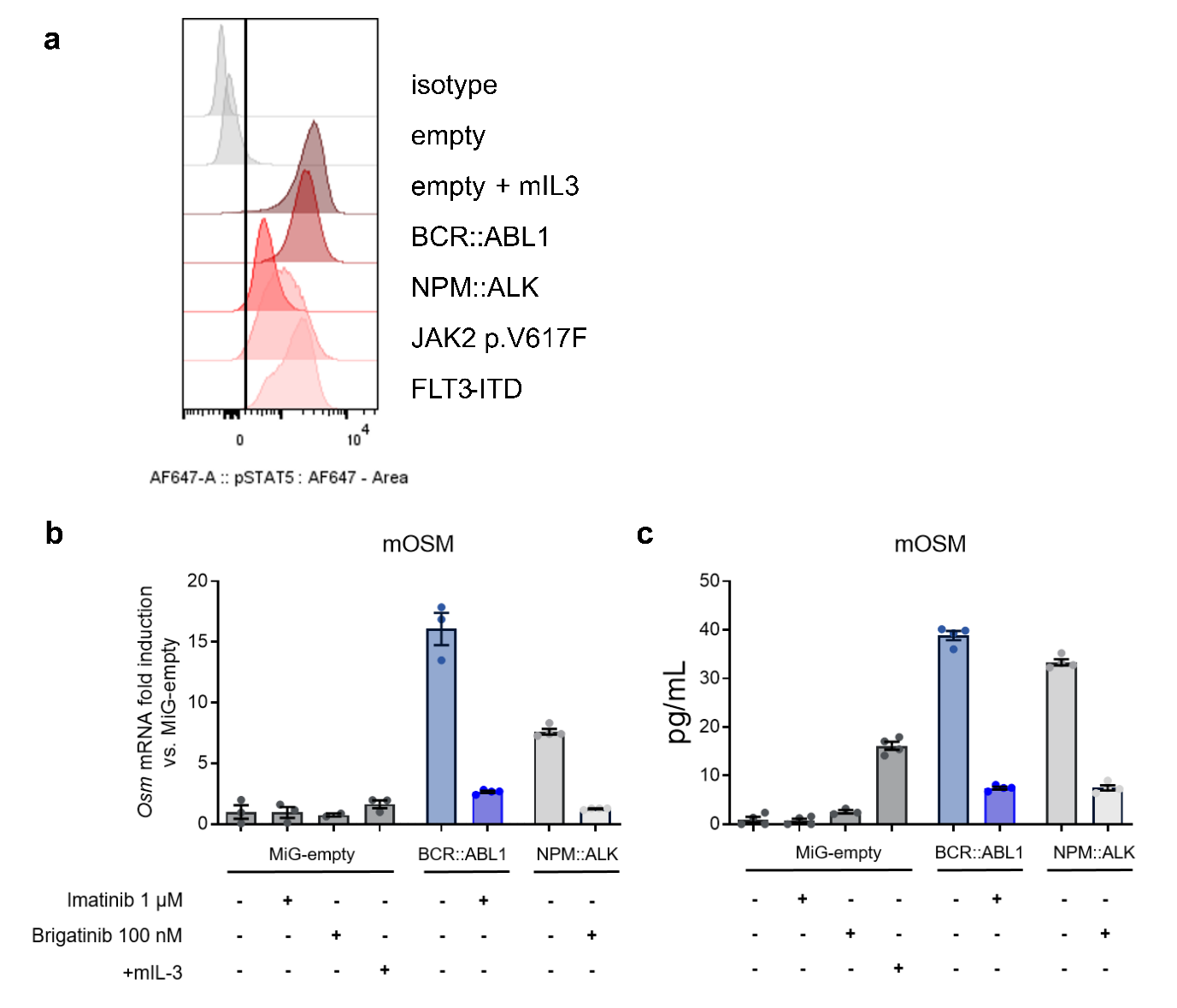
**

**Supplementary Figure 1 *Osm* transcription by STAT5-activating oncogenes correlates with OSM protein synthesis**

**(a)** Phosphoflow for pSTAT5 in MiG-empty or oncogene-transduced 32D cells starved from cytokines and serum for twelve hours. MiG-empty 32D cells were treated with mIL3 for positive control. One experiment depicted from N = 2 experiments. **(b)** 32D cells transduced with MiG-empty, BCR::ABL1 or NPM::ALK vector were starved from cytokines and serum for eight hours plus imatinib, brigatinib, or mIL3 as depicted. *Osm* expression values were normalized to *Gapdh* and empty vector (MiG-empty) expression values. One experiment depicted from N = 2 experiments. Data are represented as mean ± SEM. **(c)** Same cells as used for (b): the supernatant was collected and mOSM protein concentration was determined by ELISA. The data are presented as the means ± SEMs.

**Figure S2.**

**
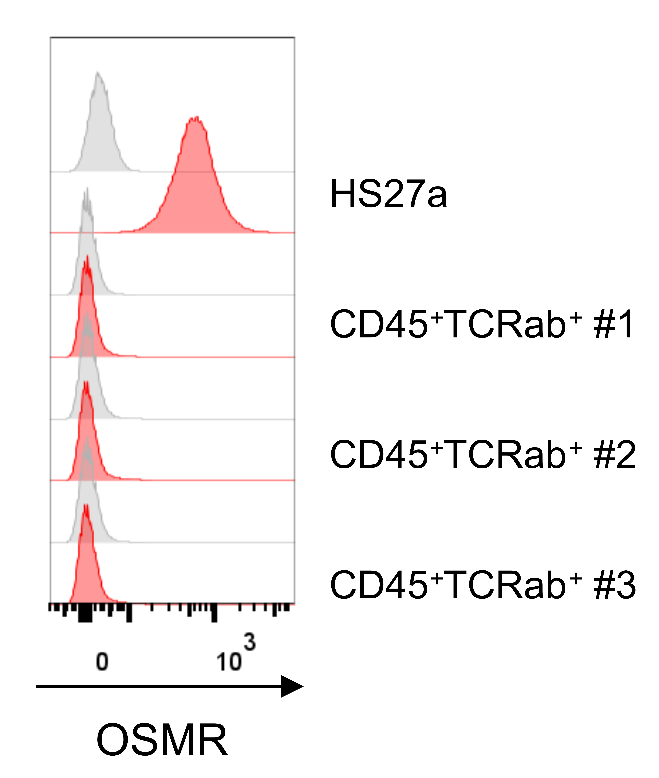
**

**Supplementary Figure 2 Absence of OSMR surface expression in circulating naïve T cells from healthy individuals**

Absence of OSMR expression on circulating CD45^+^TCRαβ^+^ T cells from healthy individuals.

**Figure S3.**

**
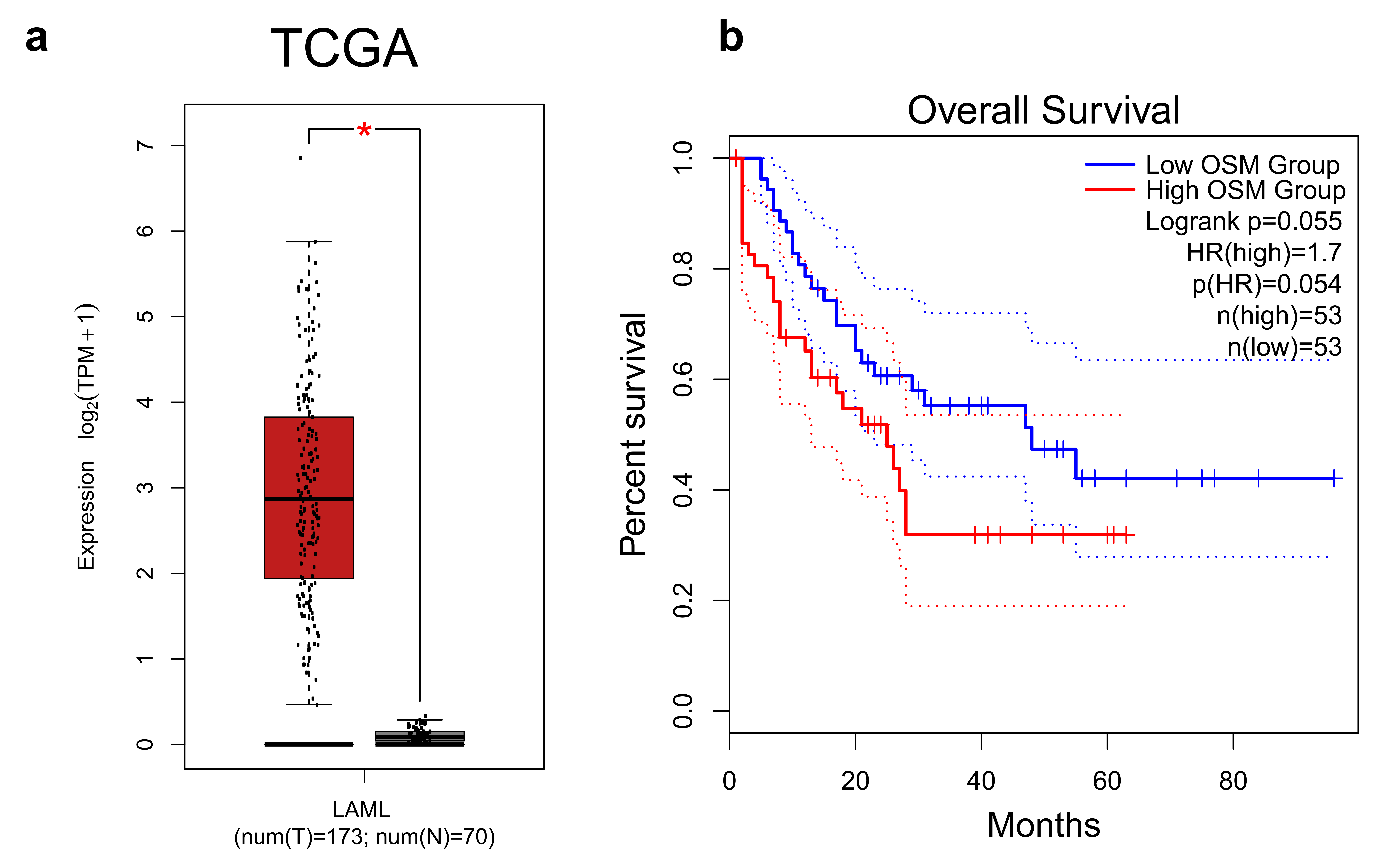
**

**Supplementary Figure 3 *OSM* expression is increased in AML patients vs. healthy individuals**

**(a)** Elevated *OSM* expression in AML patients (n=173) relative to healthy controls (n=70) within the TCGA database. Data are represented as median with interquartile range (IQR) (25th–75th percentile) within the box and whiskers extending to the minimum and maximum values within 1.5× IQR. **(b)** Improved survival for OSM^low^ vs. OSM^high^ AML patients (n=53/53).

**Figure S4.**


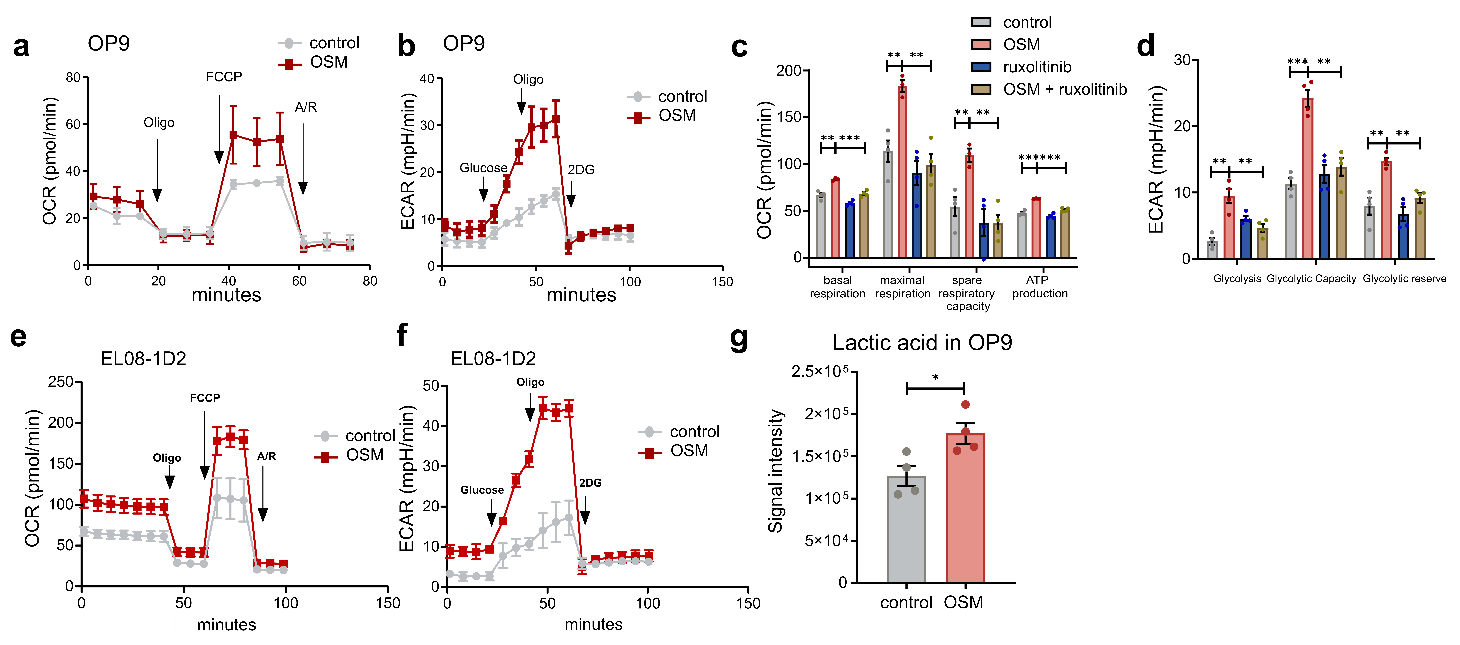


**Supplementary Figure 4 The metabolic activity in murine bone marrow stromal cells is enhanced by OSM**

**(a+b)** Mitochondrial stress test (a) and glycolysis stress test (b) of OP9 cells treated or untreated with mOSM. The data are presented as the means ± SEMs. **(c+d)** OP9 cells were co-treated with ruxolitinib which inhibited the increase of the depicted parameters in mitochondrial stress test (c) and glycolysis stress test (d). * p<0.05, ** p<0.01, ***p<0.001. The data are presented as the means ± SEMs. Abbreviations: Oligo, Oligomycin; FCCP, Carbonyl cyanide-p-trifluoromethoxyphenylhydrazone; A/R Antimycin A/ Rotenone; 2DG, 2-Deoxyglucose. **(e+f)** Mitochondrial stress test (e) and glycolysis stress test (f) of EL08-1D2 cells treated or untreated with mOSM. Abbreviations: Oligo, Oligomycin; FCCP, Carbonyl cyanide-p-trifluoromethoxyphenylhydrazone; A/R Antimycin A/ Rotenone; 2DG, 2-Deoxyglucose. The data are presented as the means ± SEMs. **(f)** Lactic acid levels assessed by low-input targeted metabolomics on (semi)polar metabolites in 5x10^5^ OP9 cells treated with mOSM (24 h, 10 ng/mL). N = 4 vs. 4. * p<0.05. The data are presented as the means ± SEMs.

**Figure S5.**

**
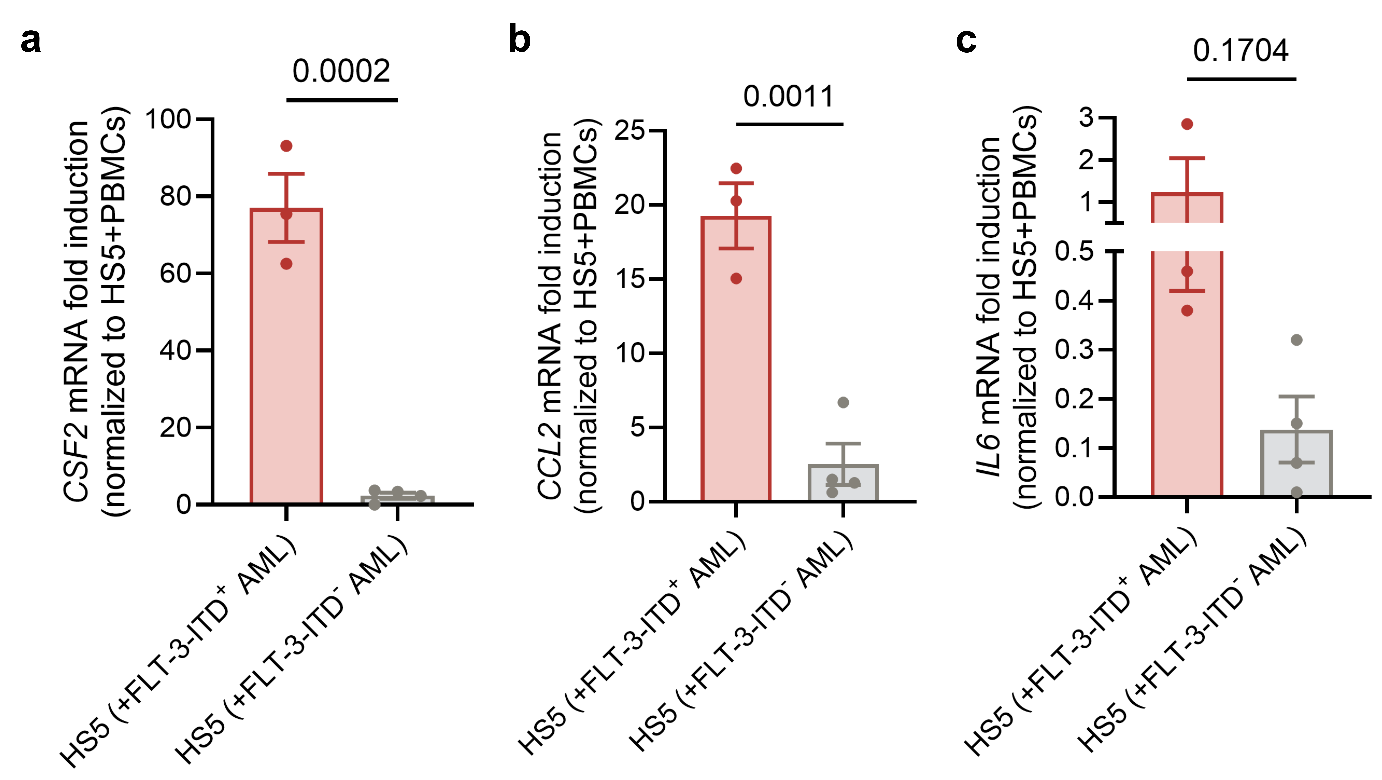
**

**Supplementary Figure 5 Elevated cytokine expression in human bone marrow stromal cells cocultured with primary FLT3-ITD^+^ AML cells**

**(a-c)** Human bone marrow stromal cells (HS5) were cocultured with primary FLT3-ITD^+^ or FLT3-ITD^-^ AML blast cells. CD90^+^ HS5 cells were sorted from coculture and cytokine transcript levels determined by qPCR. HS5 cells cocultured with FLT3-ITD^+^ AML cells showed higher transcript levels of *CSF2* (GM-CSF) (a), *CCL2* (MCP-1) (b) and a trend for higher levels of *IL6* (c). Each dot represents an individual patient. The data are presented as the means ± SEMs.

**Figure S6.**


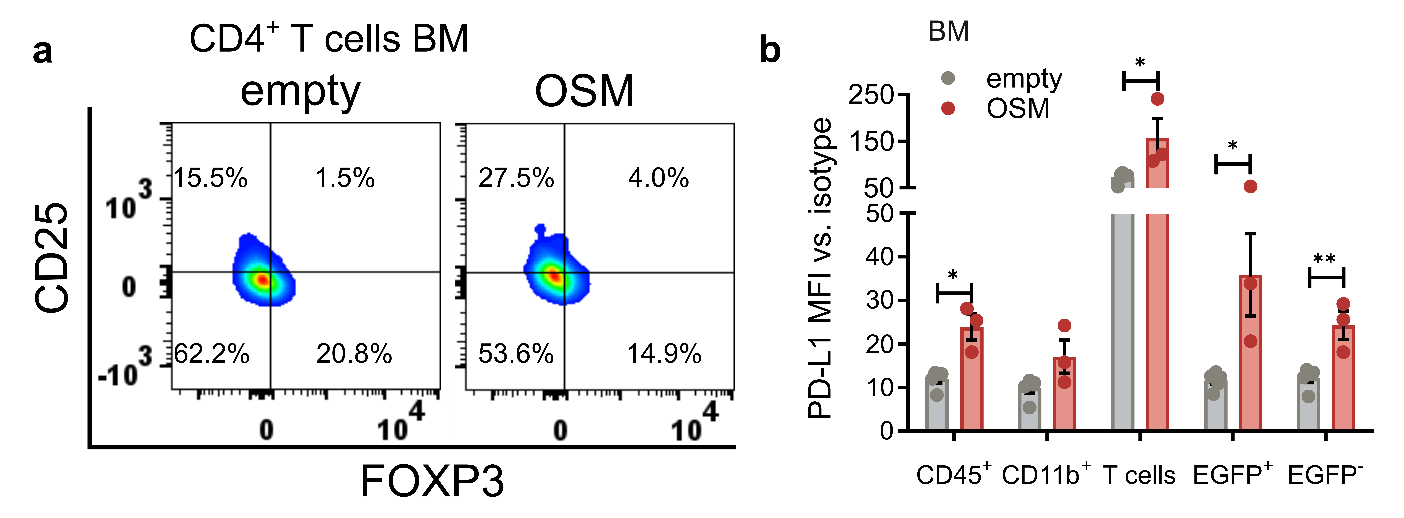


**Supplementary Figure 6 Increased percentage of regulatory T cells and elevated expression of PD-L1 on CD45^+^ cells in the BM from OSM expressing mice**

**(a)** Flow cytometric analyses of CD25^+^FOXP3^+^ CD4^+^ T cells from bone marrow (BM). **(b)** Flow cytometric analysis of PD-L1 expression on different subsets of BM derived hematopoietic cells from MiG-empty vector vs. MiG-OSM mice. Mean fluorescence intensity (MFI) of isotype control was subtracted from PD-L1 MFI values. N = 5 vs. 3. * p<0.05, ** p<0.01. The data are presented as the means ± SEMs.

**Figure S7.**

**
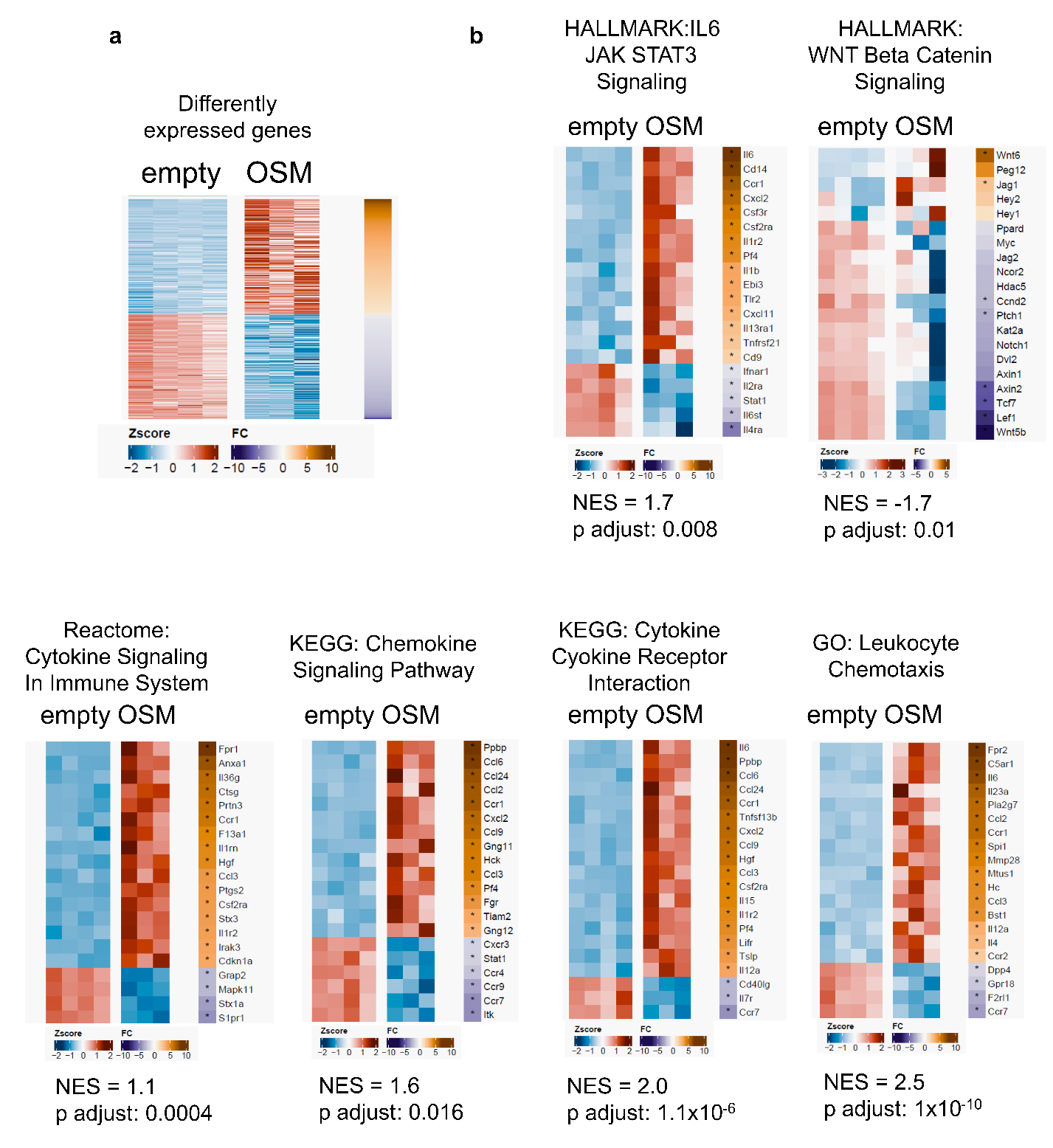
**

**Supplementary Figure 7 T cells from OSM expressing mice show marked transcriptional changes**

**(a)** Heatmaps showing the row-wise scaled gene expression (left panel) and log2 fold change between OSM and empty. Genes are ranked from high to low log2 fold change. **(b)** Gene-sets displaying the top 20 genes. Significant changes are labeled with an asterisk (“*”). Gene-set normalized enrichment score (NES) and adjusted p-value are derived from the GSEA analysis. N = 4 vs. 3.

**Figure S8.**

**
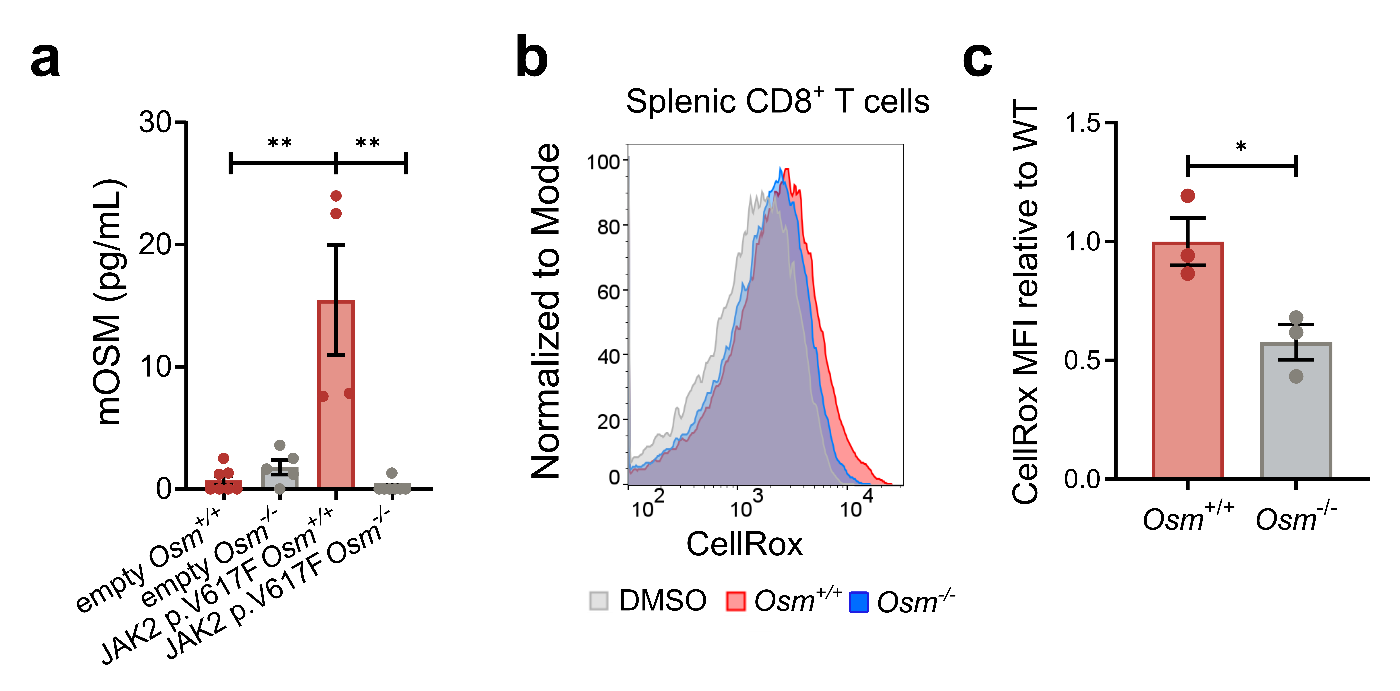
**

**Supplementary Figure 8 OSM levels are elevated in *JAK2 p.V716F Osm^+/+^* animals while T cells from *Osm* deficient JAK2 p.V617F mice display lower levels of reactive oxygen levels**

**(a)** Plasma OSM levels assessed by ELISA were elevated in *Jak2 p.V617F⁺ Osm^+/+^* mice compared to empty vector controls and *Jak2 p.V617F^+^* *Osm^⁻/⁻^* recipient mice. N = 7 vs. 3 vs. 4 vs. 7. ** p<0.01. The data are presented as the means ± SEMs. **(b+c)** CellRox staining for reactive oxygen species on CD8^+^ T cells from spleen of *Jak2 p.V617F^+^* *Osm^+/+^* vs. *Osm^-/-^* mice. N = 3 vs. 3. * p<0.05. The data are presented as the means ± SEMs.

**Figure S9.**

**
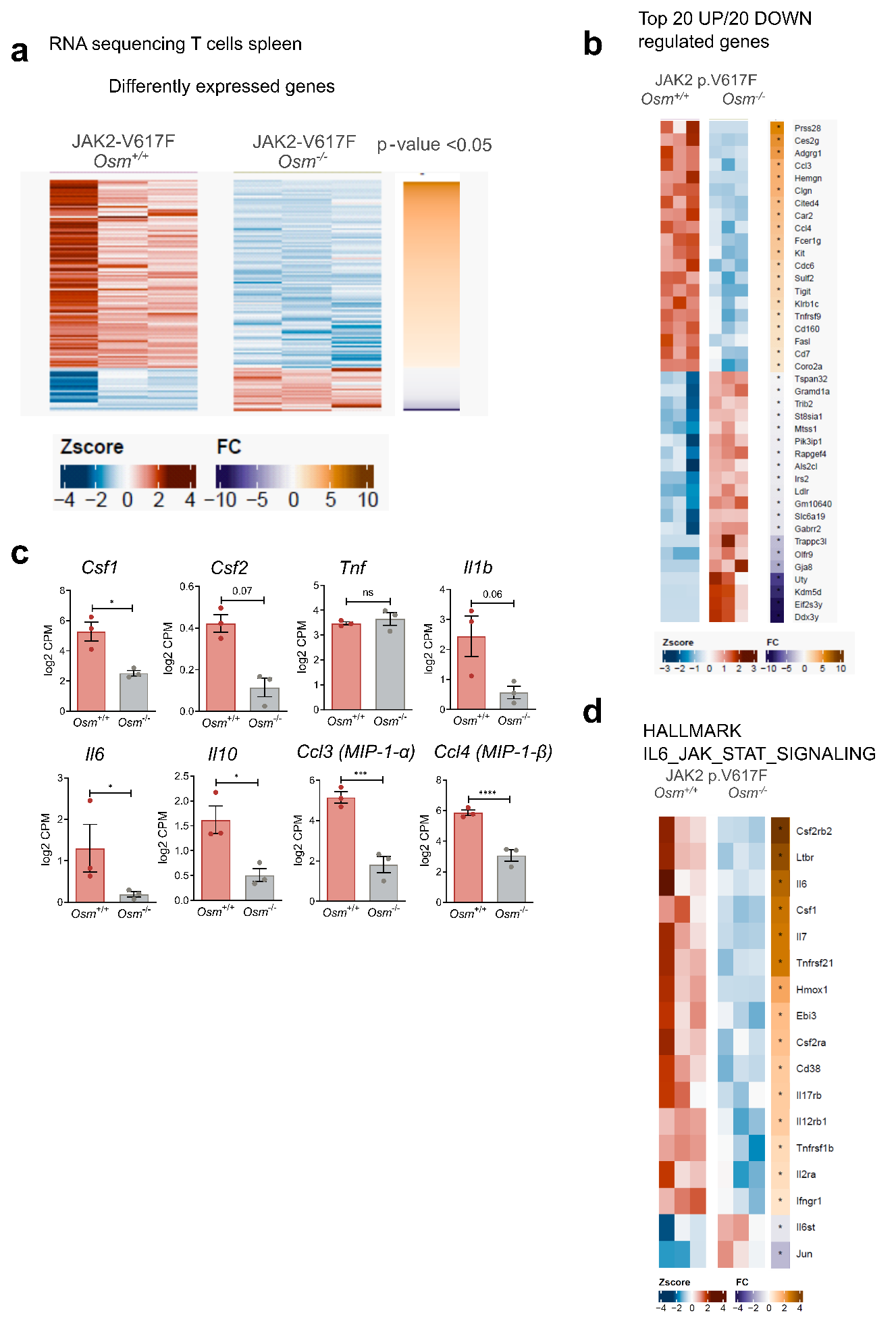
**

**Supplementary Figure 9 T cells from *Jak2 p.V617F* *Osm^+/+^* mice are transcriptionally reprogrammed**

**(a)** Heatmaps showing the row-wise scaled gene expression (left panel) and log2 fold change between *Jak2 p.V617F^+^* *Osm^+/+^* and *Jak2 p.V617F^+^* *Osm^-/-^* (right panel). Genes are ranked from high to low log2 fold change. **(b)** Heatmap showing the top 20UP and top 20 DOWN regulated genes from the same contrast. Significant changes are labeled with an asterisk (“*”). **(c)** Depiction of specific transcript levels in T cells from mice transplanted with *Jak2 p.V617F^+^ Osm^+/+^* BM vs. *Jak2 p.V617F^+^ Osm^-/-^* BM. N= 3 vs. 3, * p<0.05, *** p<0.001, *** p<0.0001. The data are presented as the means ± SEMs. **(d)** Heatmap showing the differently regulated genes within HALLMARK_IL6_JAK_STAT_Signaling. Significant changes are labeled with an asterisk (“*”).

**Figure S10.**

**
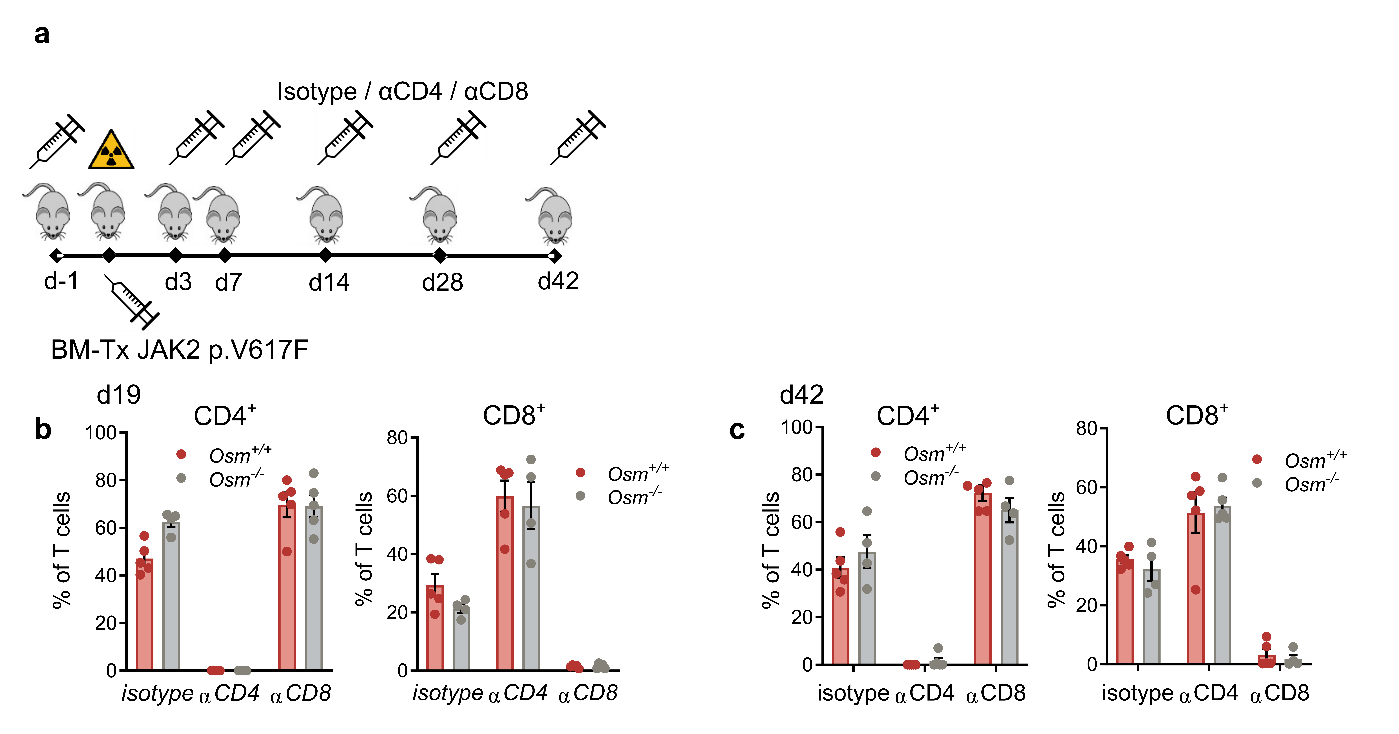
**

**Supplementary Figure 10 Anti-CD4 or -CD8 treatment effectively depletes CD4^+^ or CD8^+^ T cells**

**(a)** Illustration depicting the administration schedule of the anti-CD4, anti-CD8, or isotype treatments in mice. Treatment was started three days after transplantation of *Jak2 p.V617F^+^ Osm^+/+^* or *Jak2 p.V617F^+^ Osm^-/-^* BM, followed by subsequent administrations as outlined. N= 5 for each genotype and treatment. **(b+c)** Flow cytometric evaluation of CD4^+^ (left) and CD8^+^ (right) T cells in the peripheral blood 19 (b) or 42 (c) days after *Jak2 p.V617F Osm^+/+^* vs. *Osm^-/-^* BM transplantation. Distinct antibody clonotypes targeting different epitopes were employed for either depletion or FACS detection of CD4 and CD8, respectively. Each data point represents one animal. The data are presented as the means ± SEMs.

**Figure S11.**

**
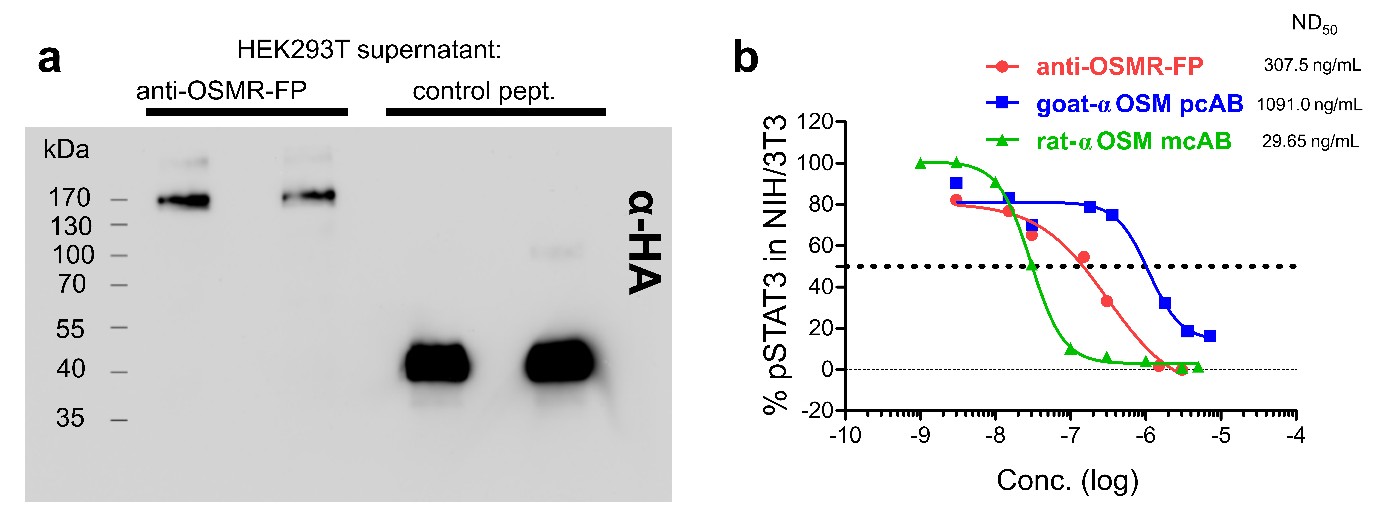
**

**Supplementary Figure 11 Testing the anti-OSM receptor fusion protein (anti-OSMR-FP) and monoclonal anti-OSM antibody**

**(a)** Immunoblot against HA on the purified anti-OSMR-FP (left two bands) vs. control peptide (right two bands) from supernatant of transgenic HEK293T cells. An uncropped version of the blot is available below. **(b)** Flow cytometric evaluation of pSTAT3 levels in NIH/3T3 cells upon OSM treatment plus anti-OSMR-FP, goat-derived polyclonal or rat-derived monoclonal anti-mouse OSM (αmOSM) antibody at different concentrations as indicated.

**Table S1.**

|  | **Specimen** | **Age** | **Sex** | **Disease** | **Mutation** | **First Diagnosis** | **Remission Status** | **Material** | **Column1** | **hOSM fc** |  |
| --- | --- | --- | --- | --- | --- | --- | --- | --- | --- | --- | --- |
|  | MPN1 | 73 | M | CML | BCR::ABL1, p210, b3a2 | 05/2015 | first diagnosis | PB |  | 2.53 |  |
|  | MPN2 | 58 | F | CML | BCR::ABL1, p210 | 10/2015 | first diagnosis | PB |  | 1.64 |  |
|  | MPN3 | 55 | F | CML | BCR::ABL1, p210, b2a2 | 01/2018 | first diagnosis | PB |  | 7.22 |  |
|  | MPN4 | 50 | F | CML | BCR::ABL1, p210, b2a2 | 04/2018 | first diagnosis | PB |  | 4.18 |  |
|  | MPN5 | 67 | F | CML | BCR::ABL1, p210, b3a2 | 06/2018 | first diagnosis | PB |  | 8.69 |  |
|  | MPN6 | 28 | M | CML | BCR::ABL1, p210, a2b2 | 07/2018 | first diagnosis | PB |  | 3.81 |  |
|  | MPN7 | 59 | F | CML | BCR::ABL1, p210 | 11/2018 | first diagnosis | PB |  | 15.07 |  |
|  | MPN8 | 38 | M | CML | BCR::ABL1, p210, e14a2 | 01/2019 | first diagnosis | PB |  | 1.61 |  |
|  | MPN9 | 33 | M | CML | BCR::ABL1, p210 | 01/2019 | first diagnosis | PB |  | 6.65 |  |
|  | MPN10 | 78 | M | CML | BCR::ABL1, p210, e14a2 | 02/2019 | first diagnosis | PB |  | 10.08 |  |
|  | MPN11 | 54 | M | CML | BCR::ABL1, p210, e13a2 | 02/2019 | first diagnosis | PB |  | 6.04 |  |
|  | MPN12 | 58 | F | CML | BCR::ABL1, p210, e14a2 | 02/2019 | first diagnosis | PB |  | 19.78 |  |
|  | MPN13 | 24 | M | CML | BCR::ABL1, p210, e13a2 | 03/2019 | first diagnosis | PB | n=13 | 4.58 |  |
|  |  |  |  |  |  |  |  |  |  |  |  |
|  | MPN14 | 38 | M | CML | BCR::ABL1, p210 | 06/2015 | first diagnosis | BM |  | 0.6 |  |
|  | MPN15 | 71 | M | CML | BCR::ABL1, p210 | 09/2015 | first diagnosis | BM |  | 9.08 |  |
|  | MPN16 | 66 | M | CML | BCR::ABL1, p210 | 10/2015 | first diagnosis | BM |  | 19.95 |  |
|  | MPN17 | 59 | M | CML | BCR::ABL1, p210, b2a2 | 06/2016 | first diagnosis | BM |  | 2.74 |  |
|  | MPN18 | 41 | M | CML | BCR::ABL1, p210, b2a2 | 08/2017 | first diagnosis | BM |  | 12.76 |  |
|  | MPN19 | 58 | M | CML | BCR::ABL1, p210 | 06/2018 | first diagnosis | BM |  | 16.69 |  |
|  | MPN20 | 67 | F | CML | BCR::ABL1, p210, b3a2 | 06/2018 | first diagnosis | BM |  | 25.78 |  |
|  | MPN21 | 59 | F | CML | BCR::ABL1, p210 | 11/2018 | first diagnosis | BM |  | 6.26 |  |
|  | MPN22 | 38 | M | CML | BCR::ABL1, p210, e14a2 | 01/2019 | first diagnosis | BM |  | 6.78 |  |
|  | MPN23 | 54 | M | CML | BCR::ABL1, p210, e13a2 | 02/2019 | first diagnosis | BM |  | 4.4 |  |
|  | MPN24 | 58 | F | CML | BCR::ABL1, p210, e14a2 | 02/2019 | first diagnosis | BM | n=11 | 6.96 |  |
|  |  |  |  |  |  |  |  |  |  |  |  |

**Table S2.**

|  |  |  |  |  |  |  |  |  |  |  |  |  |  |
| --- | --- | --- | --- | --- | --- | --- | --- | --- | --- | --- | --- | --- | --- |
|  |  |  |  |  |  |  |  |  |  |  | huOSM (pg/mL) | |  |
|  | **Specimen** | **Age** | **Sex** | **FLT3-ITD status** | **Other Mutations** | **Cytogenetics** | **First Diagnosis** | **Treatment** | **Remission Status** | **Column1** | **Value 1** | **Value 2** |  |
|  | AML1 | 43 | M | Positive (high) | NPM1 Mut. (Typ A), DNMT3A | trisomy 5, trisomy 8 | 2023 | 7+3, 2 x allo-Tx, gilteritinib, sorafenib | relapse |  | 144.2198 | 142.0496 |  |
|  | AML2 | 38 | M | Positive (high) | high WT1 expression | NUB98 | 2023 | 7+3, sorafenib, allo-Tx, gilteritinib | first diagnosis |  | 674.5551 | 637.8874 |  |
|  | AML3 | 79 | F | Positive (high) | ASXL1 | deletion 13q14/DLEU | 2024 | decitabine, venetoclax | first diagnosis |  | 306.5131 | 333.6115 |  |
|  | AML4 | 53 | F | Positive (low) | DNMT3A R8882c | - | 2022 | 7+3, sorafenib, allo-Tx, gilteritinib | relapse |  | 280.1261 | 298.6861 |  |
|  | AML5 | 48 | M | Positive (high) | BCOR, DNMT3A, IDH2 | trisomy 8, complex caryotype | 2023 | 7+3 + sorafenib, allo-Tx, decitabine + venetoclax | blast persistence upon therapy | n=5 | 31.23273 | 33.01738 |  |
|  |  |  |  |  |  |  |  |  |  |  |  |  |  |
|  | AML6 | 36 | M | Positive (high) | NUP98 adn NUPS98-NSD1 rearrangements | deletion in NUP98 | 2021 | 7+3 + sorafenib, 2 x allo-Tx | Complete remission |  | 9.647289 | 9.90981 |  |
|  | AML7 | 59 | F | Positive (low) | - | - | 2023 | 7+3, allo-Tx, sorafenib | Complete remission |  | 54.17505 | 50.34438 |  |
|  | AML8 | 49 | F | Positive (high) | NPM1 mut | - |  | 7+3 + sorafenib, allo-Tx, sorafenib | Complete remission |  | 33.00469 | 37.89504 |  |
|  | AML9 | 72 | F | Positive (high) | NPM1B mut., DNMT3A mut CEBPA wt | - | 2018 | MICE, mini-ICE, allo-Tx | Complete remission |  | 0 | 0 |  |
|  | AML10 | 72 | F | Positive (low) | NPM1-A, TET2, FLT3-TKD | - | 2018 | acacitidine, allo-Tx | Complete remission |  | 107.3096 | 103.9168 |  |
|  | AML11 | 72 | M | Positive | - | - | 2015 | 7+3, S-HAM, sorafenib, allo-Tx | Complete remission | n=6 | 0 | 0 |  |
|  |  |  |  |  |  |  |  |  |  |  |  |  |  |
|  | AML12 | 54 | M | Negative | RUNX1-RUNX1T1 | CBFB-MYH11, Inv(16) | 2022 | 7+3, consolidation, decitabine + venetoclax, allo-Tx | Complete remission |  | 57.89501 | 53.65962 |  |
|  | AML13 | 72 | F | Negative | SRSF2, TET2, RUNX1 | del (20)(q11q13) | 2023 | decitabine + venetoclax, allo-Tx | Complete remission |  | 33.97486 | 35.41973 |  |
|  | AML14 | 72 | M | Negative | DNMT3A, CUX1, TET2 (2x), KRAS, STAG2 | cMYC amplification | 2022 | 7+3, allo-Tx | Complete remission |  | 1.072778 | 0.862918 |  |
|  | AML15 | 76 | F | Negative | NRAS, PTPN11 | - | 2023 | azacitidine + venetoclax, allo-Tx, sabatolimab + azacitidine | Complete remission |  | 0 | 0 |  |
|  | AML16 | 69 | M | Negative | ASXL1, PHF6 | deletion 21q22/5'RUNX1 | 2023 | decitabin + venetoclax, allo-Tx | Complete remission |  | 0 | 0.478481 |  |
|  | AML17 | 49 | F | Negative | TP53 | - | 2023 | allo-Tx | Complete remission |  | 0 | 0 |  |
|  | AML18 | 71 | M | Negative | RUNX1-rearrangement | trisomy 8 | 2020 | decitabine + venetoclax, haplo-identical Tx | Complete remission |  | 10.41149 | 9.194268 |  |
|  | AML19 | 29 | M | Negative | CBFB-MYH11, inversion 16 type A | t(8;11) (p23;q21), Inversion 16 (p13.1q22), +22 | 2022 | 7+3, allo-Tx | Complete remission |  | 1.574576 | 1.29442 |  |
|  | AML 20 | 63 | M | Negative | EVI1-rearrangement | 46 XY, ins(3;3) (p11q21;q26),-7,+8 | 2008 | 7+3, allo-Tx, decitabine+ venetoclax | Complete remission |  | 4.905569 | 6.605761 |  |
|  | AML21 | 72 | F | Negative | CBFB-MYH11, Inversion 16 type A | Inversion (16) (p13.1;q22) | 2022 | 7+3, allo-Tx, decitabine+ venetoclax | Complete remission |  | 33.00469 | 37.89504 |  |
|  | AML22 | 72 | F | Negative | BCOR, BCORL1, DNMT3A, SF3B1, STAG2, TET2 | - | 2019 | MICE, allo-Tx | Complete remission |  | 50.07531 | 46.47788 |  |
|  | AML23 | 63 | M | Negative | - | complex rearrangement | 2005 | 7+3, allo-Tx | Complete remission |  | 176.9903 | 178.6045 |  |
|  | AML24 | 51 | F | Negative | ASXL1, IDH2, SRSF2 | trisomy 8 | 2023 | allo-Tx | Complete remission |  | 199.6793 | 183.0668 |  |
|  | AML25 | 52 | F | Negative | NPM1 Typ 1 A, TP53 | - | 2023 | 7+3+gemtuzumab-ozogamicin, allo-Tx | Complete remission |  | 17.72936 | 17.19685 |  |
|  | AML26 | 49 | M | Negative | TP53-mutation | deletion 5q31/5q33, deletion 7q22/7q31 | 2023 | 7+3, allo-Tx | Complete remission |  | 0 | 0 |  |
|  | AML27 | 50 | F | Negative | WT-1 expression | - | 2000 | AraC, DNR, VP-16, allo-Tx | Complete remission |  | 0.874912 | 0.158052 |  |
|  | AML28 | 72 | F | Negative | BCOR, IDH1, SF3B1, TP53 | 5q- | 2023 | lenalidomide, vidaza, venetoclax; allo-Tx | Complete remission |  | 5.731308 | 7.02925 |  |
|  | AML29 | 61 | F | Negative | KMT2A-MLLT3 rearrangement, t(9,11) | - | 2021 | decitabine+ venetoclax, allo-Tx | Complete remission |  | 190.6351 | 191.1693 |  |
|  | AML30 | 66 | M | Negative | CEBPA-Mutation (homozygous) | - | 2019 | 7+3, HAM, AraC, allo-Tx | Complete remission |  | 0 | 0 |  |
|  | AML31 | 58 | M | Negative | WT1 high expression, TP53 | Deletion 5q31/5q33/EGR1/RPS14, Deletion 21q22/RUNX1, Deletion 7q22/7q31/KMT2E/MET | 2021 | vyxeos liposomal, allo-Tx | Complete remission |  | 0 | 0 |  |
|  | AML32 | 66 | M | Negative | DDX41, RUNX1, SRSF2 | - | 2022 | allo-Tx | Complete remission |  | 63.99431 | 67.14344 |  |
|  | AML33 | 38 | M | Negative | FLT3-ITDlow, BCOR, BORL1, RUNX1 | - | 2019 | 7+3+gemzuzumab-ozogamicin, allo-Tx, decitabine+ venetoclax | Complete remission |  | 134.41 | 149.88 |  |
|  | AML34 | 46 | F | Negative | NRAS Mut | monosomy 7 | 2023 | vyxeos, decitabine+venetoclax, allo-Tx | Complete remission | n=23 | 71.04522 | 76.40875 |  |
|  |  |  |  |  |  |  |  |  |  |  |  |  |  |
|  |  |  |  |  |  |  |  |  |  |  |  |  |  |
|  | AML35 | 61 | M | Negative | IDH2 | - | 2016 | ICE, allo-Tx, decitabine+ venetoclax | relapse |  | 96.60415 | 72.07413 |  |
|  | AML36 | 38 | F | Negative | TP53 double-hit mutations, PTPN11 < 5%, EZH2 | complex karyotype | 2023 | allo-Tx | relapse |  | 107.7911 | 197.411 |  |
|  | AML37 | 62 | F | Negative | WT1, KRAS, DNMT3A, KMT2A::KNL1 fusion | t(11;15)(q23.3;q15.1) | 2023 | allo-Tx | relapse |  | 25.18223 | 22.90242 |  |
|  | AML38 | 78 | M | Negative | IDH1, JAK2 p.V617F, RUNX1, SRSF2 | - | 2021 | decitabin/venetoclax, azacitidine/venetoclax | relapse |  | 79.16805 | 110.5044 |  |
|  | AML39 | 54 | M | Negative | NRAS, TP53 | 5q del, 7q del, 17p13 del | 2023 | decitabin/venetoclax +/- ATRA/placebo, allo-Tx | relapse |  | 119.4098 | 173.5622 |  |
|  | AML40 | 61 | F | Negative | DNMT3A, NPM1 type A | - | 2023 | decitabine+venetoclax, allo-Tx | relapse | n=6 | 2.093581 | 2.646 |  |

**Supplementary Text**

**Sequences of oncogenes**

JAK2 p.V617F

atgggaatggcctgccttacgatgacagaaatggagggaacatccacctcttctatatatcagaatggtgatatttctggaaatgccaattctatagatccagttcttcaggtgtatctttaccattcccttgggaaatctgaggcagattatctgacctttccatctggggagtatgttgcagaagaaatctgtattgctgcttctaaagcttgtggtatcacacctgtgtatcataatatgtttgctttaatgagtgaaacagaaaggatctggtatccacccaaccatgtcttccatatagatgagtcaaccaggcataatgtactctacagaataagattttactttcctcgttggtattgcagtggcagcaacagagcctatcggcatggaatatctcgaggtgctgaagctcctcttcttgatgactttgtcatgtcttacctctttgctcagtggcggcatgattttgtgcacggatggataaaagtacctgtgactcatgaaacacaggaagaatgtcttgggatggcagtgttagatatgatgagaatagccaaagaaaacgatcaaaccccactggccatctataactctatcagctacaagacattcttaccaaaatgtattcgagcaaagatccaagactatcatattttgacaaggaagcgaataaggtacagatttcgcagatttattcagcaattcagccaatgcaaagccactgccagaaacttgaaacttaagtatcttataaatctggaaactctgcagtctgccttctacacagagaaatttgaagtaaaagaacctggaagtggtccttcaggtgaggagatttttgcaaccattataataactggaaacggtggtattcagtggtcaagagggaaacataaagaaagtgagacactgacagaacaggatttacagttatattgcgattttcctaatattattgatgtcagtattaagcaagcaaaccaagagggttcaaatgaaagccgagttgtaactatccataagcaagatggtaaaaatctggaaattgaacttagctcattaagggaagctttgtctttcgtgtcattaattgatggatattatagattaactgcagatgcacatcattacctctgtaaagaagtagcacctccagccgtgcttgaaaatatacaaagcaactgtcatggcccaatttcgatggattttgccattagtaaactgaagaaagcaggtaatcagactggactgtatgtacttcgatgcagtcctaaggactttaataaatattttttgacttttgctgtcgagcgagaaaatgtcattgaatataaacactgtttgattacaaaaaatgagaatgaagagtacaacctcagtgggacaaagaagaacttcagcagtcttaaagatcttttgaattgttaccagatggaaactgttcgctcagacaatataattttccagtttactaaatgctgtcccccaaagccaaaagataaatcaaaccttctagtcttcagaacgaatggtgtttctgatgtaccaacctcaccaacattacagaggcctactcatatgaaccaaatggtgtttcacaaaatcagaaatgaagatttgatatttaatgaaagccttggccaaggcacttttacaaagatttttaaaggcgtacgaagagaagtaggagactacggtcaactgcatgaaacagaagttcttttaaaagttctggataaagcacacagaaactattcagagtctttctttgaagcagcaagtatgatgagcaagctttctcacaagcatttggttttaaattatggagtatgtttctgtggagacgagaatattctggttcaggagtttgtaaaatttggatcactagatacatatctgaaaaagaataaaaattgtataaatatattatggaaacttgaagttgctaaacagttggcatgggccatgcattttctagaagaaaacacccttattcatgggaatgtatgtgccaaaaatattctgcttatcagagaagaagacaggaagacaggaaatcctcctttcatcaaacttagtgatcctggcattagtattacagttttgccaaaggacattcttcaggagagaataccatgggtaccacctgaatgcattgaaaatcctaaaaatttaaatttggcaacagacaaatggagttttggtaccactttgtgggaaatctgcagtggaggagataaacctctaagtgctctggattctcaaagaaagctacaattttatgaagataggcatcagcttcctgcaccaaagtgggcagaattagcaaaccttataaataattgtatggattatgaaccagatttcaggccttctttcagagccatcatacgagatcttaacagtttgtttactccagattatgaactattaacagaaaatgacatgttaccaaatatgaggataggtgccctAgggttttctggtgcctttgaagaccgggatcctacacagtttgaagagagacatttgaaatttctacagcaacttggcaagggtaattttgggagtgtggagatgtgccggtatgaccctctacaggacaacactggggaggtggtcgctgtaaaaaagcttcagcatagtactgaagagcacctaagagactttgaaagggaaattgaaatcctgaaatccctacagcatgacaacattgtaaagtacaagggagtgtgctacagtgctggtcggcgtaatctaaaattaattatggaatatttaccatatggaagtttacgagactatcttcaaaaacataaagaacggatagatcacataaaacttctgcagtacacatctcagatatgcaagggtatggagtatcttggtacaaaaaggtatatccacagggatctggcaacgagaaatatattggtggagaacgagaacagagttaaaattggagattttgggttaaccaaagtcttgccacaagacaaagaatactataaagtaaaagaacctggtgaaagtcccatattctggtatgctccagaatcactgacagagagcaagttttctgtggcctcagatgtttggagctttggagtggttctgtatgaacttttcacatacattgagaagagtaaaagtccaccagcggaatttatgcgtatgattggcaatgacaaacaaggacagatgatcgtgttccatttgatagaacttttgaagaataatggaagattaccaagaccagatggatgcccagatgagatctatatgatcatgacagaatgctggaacaataatgtaaatcaacgcccctcctttagggatctagctcttcgagtggatcaaataagggataacatggctggatga

BCR::ABL1 (p185, e14a2 (b3a2))

atggtggacccggtgggcttcgcggaggcgtggaaggcgcagttcccggactcagagcccccgcgcatggagctgcgctcagtgggcgacatcgagcaggagctggagcgctgcaaggcctccattcggcgcctggagcaggaggtgaaccaggagcgcttccgcatgatctacctgcagacgttgctggccaaggaaaagaagagctatgaccggcagcgatggggcttccggcgcgcggcgcaggcccccgacggcgcctccgagccccgagcgtccgcgtcgcgcccgcagccagcgcccgccgacggagccgacccgccgcccgccgaggagcccgaggcccggcccgacggcgagggttctccgggtaaggccaggcccgggaccgcccgcaggcccggggcagccgcgtcgggggaacgggacgaccggggaccccccgccagcgtggcggcgctcaggtccaacttcgagcggatccgcaagggccatggccagcccggggcggacgccgagaagcccttctacgtgaacgtcgagtttcaccacgagcgcggcctggtgaaggtcaacgacaaagaggtgtcggaccgcatcagctccctgggcagccaggccatgcagatggagcgcaaaaagtcccagcacggcgcgggctcgagcgtgggggatgcatccaggcccccttaccggggacgctcctcggagagcagctgcggcgtcgacggcgactacgaggacgccgagttgaacccccgcttcctgaaggacaacctgatcgacgccaatggcggtagcaggcccccttggccgcccctggagtaccagccctaccagagcatctacgtcgggggcatgatggaaggggagggcaagggcccgctcctgcgcagccagagcacctctgagcaggagaagcgccttacctggccccgcaggtcctactccccccggagttttgaggattgcggaggcggctataccccggactgcagctccaatgagaacctcacctccagcgaggaggacttctcctctggccagtccagccgcgtgtccccaagccccaccacctaccgcatgttccgggacaaaagccgctctccctcgcagaactcgcaacagtccttcgacagcagcagtccccccacgccgcagtgccataagcggcaccggcactgcccggttgtcgtgtccgaggccaccatcgtgggcgtccgcaagaccgggcagatctggcccaacgatggcgagggcgccttccatggagacgcagatggctcgttcggaacaccacctggatacggctgcgctgcagaccgggcagaggagcagcgccggcaccaagatgggctgccctacattgatgactcgccctcctcatcgccccacctcagcagcaagggcaggggcagccgggatgcgctggtctcgggagccctggagtccactaaagcgagtgagctggacttggaaaagggcttggagatgagaaaatgggtcctgtcgggaatcctggctagtctagagaggccagtggcatctgactttgagccccagggtctcagcgaagcagctcgatggaactccaaggaaaaccttcttgctgggcccagtgaaaatgaccccaacctttttgtggcactctatgattttgtggccagtggagataacactctcagcatcactaaaggtgaaaagctccgggtcttgggttataatcacaatggggaatggtgtgaagcccaaacgaaaaatggccaaggatgggtcccaagcaactacatcacccccgtcaacagcctggagaaacattcctggtatcatggccctgtatctcggaatgctgctgagtatctgctgagcagcggaatcaacggcagcttcttagtgcgggagagtgagagtagccctggccagagatccatctcgctgcggtatgaagggagggtgtaccactacaggatcaacactgcctctgatggcaagctgtacgtgtcctccgagagccgcttcaacactctggctgagttagttcaccatcactccacggtggctgatggcctcatcaccacactccactacccagctcccaagcgcaacaagcccactatctacggtgtgtcccccaactacgacaagtgggaaatggagcgcaccgacatcaccatgaagcacaagttgggtggaggccagtacggggaggtgtacgagggcgtttggaagaagtacagcctcactgtggccgtgaagaccttgaaggaggacaccatggaggtggaggagttcctgaaggaagcggcggtgatgaaggagatcaaacaccctaacctggtgcagctgctaggggtgtgtacccgggaaccaccattctacataatcactgagttcatgacctatgggaacctgctggactacctgagggagtgtaaccggcaggaggtgagcgccgtggtactgctctacatggccacacagatctcatcagccatggagtacttggagaagaagaacttcatccacagagaccttgctgcccggaactgcctggtaggggaaaaccacttggtgaaggtggctgattttggcctgagcaggttgatgacaggggacacctacacggcccatgctggagccaaattccccatcaaatggaccgcacctgagagcctggcctacaacaagttctccatcaagtcggacgtgtgggcatttggagtattgctctgggagattgctacctatggcatgtcaccttacccgggaattgacctgtctcaggtttatgagctgctggaaaaagactaccgcatggagcgccctgaaggctgcccggagaaggtctacgagctcatgcgagcatgttggcagtggaacccctctgaccggccctcctttgctgaaatccaccaagcctttgaaaccatgttccaggaatccagtatctcagatgaggtggagaaggagctggggaaacgaggcacgagaggaggtgctgggagtatgctgcaggccccagagctgcccaccaagaccagaacctgcaggagagcagctgagcagaaagatgcgcctgacacccctgagctgctccacacgaagggcctgggagaaagcgatgcactggacagtgagcctgctgtatcgccactgcttcctcggaaagagcgcgggcccccagacggcagcctaaatgaagatgagcgccttctccccagagacagaaagaccaacctgttcagcgctttgatcaagaagaagaagaaaatggcgccgacgccccctaagcgcagcagttccttccgagagatggatggccagccagaccgcagaggggctagtgaggatgacagcagggaactctgcaatggaccaccagctctcacctcagacgcagcagagcctaccaagtccccaaaggccagcaatggggctggcgtccctaatggagccttccgggagccgggcaactcaggcttccgttctccccacatgtggaaaaagtccagcacactgaccgggagccgcctggctgctgccgaagaggagagcggcatgagctccagtaagcgcttcctgcgttcttgttcggcctcctgcatgccccatggggcaagggacacagagtggcggtcggtcacgctgcctcgagacctgccgtctgctggcaagcagtttgactcatccacctttggagggcacaaaagcgaaaagccagctctgcctcggaaacgcaccagtgagagcaggtctgagcaggtggccaaaagcacggcgatgcccctccccggctggttgaagaagaacgaggaggctgctgaagaaggcttcaaagacacagaatccagccctggctccagccctcccagcttgactcccaaactcctccgcaggcaggtcactgcctctccttcctctggcctctctcacaaggaagaggccaccaagggcagtgcctcaggcatggggactccggccactgcagagccagcaccccccagcaacaaagtgggcctcagcaaggcctcctctgaggagatgcgcgtaaggaggcacaagcacagctcggagtccccagggagagacaaggggcgactggctaagctcaagcctgccccgccgcctcctcctgcctgcacaggaaaagcaggcaagcccgcacagagccccagccaagaggccggggaggcaggggggcccacaaagacaaaatgcacgagtctggctatggatgctgtgaacactgaccccaccaaggccggcccacctggagaaggactgagaaagcctgtgcccccatctgtgccaaagccccagtcgacggctaagcctccagggactcccaccagcccggtctccaccccctccacagcaccagctccttcacccctggctggggaccagcagccatcttctgccgccttcatccccctcatatcaacccgtgtgtctcttaggaagacccgccagccgccagagcgcattgccagtggcaccatcaccaagggtgtggttctggacagtactgaggccctgtgccttgccatctcccggaactcagagcagatggccagccacagtgctgtactggaggctggcaagaacctgtacactttctgtgtgagctatgtggactctatccagcagatgaggaacaagtttgccttccgtgaggctatcaacaagctggagagcaacctccgagagctgcagatctgccctgccacagcctccagtgggccagctgccacccaagacttcagcaagctgctcagctctgtgaaggagatcagcgacattgtccggagg

FLT3-ITD

ATGCGGGCGTTGGCGCAGCGCAGCGACCGGCGGCTGCTGCTACTTGTTGTTTTGTCAGTAATGATTCTTGAGACCGTTACAAACCAAGACCTGCCTGTGATCAAGTGTGTTTTAATCAGTCATGAGAACAATGGCTCATCAGCGGGAAAGCCATCATCGTACCGAATGGTGCGAGGATCCCCAGAAGACCTCCAGTGTACCCCGAGGCGCCAGAGTGAAGGGACGGTATATGAAGCGGCCACCGTGGAGGTGGCCGAGTCTGGGTCCATCACCCTGCAAGTGCAGCTCGCCACCCCAGGGGACCTTTCCTGCCTCTGGGTCTTTAAGCACAGCTCCCTGGGCTGCCAGCCGCACTTTGATTTACAAAACAGAGGAATCGTTTCCATGGCCATCTTGAACGTGACAGAGACCCAGGCAGGAGAATACCTACTCCATATTCAGAGCGAAGCCGCCAACTACACAGTACTGTTCACAATGAATGTAAGAGATACACAGCTGTATGTGCTAAGGAGACCTTACTTTAGGAAGATGGAAAACCAGGATGCACTGCTCTGCATCTCCGAGGGTGTTCCGGAGCCCACTGTGGAGTGGGTGCTCTGCAGCTCCCACAGGGAAAGCTGTAAAGAAGAAGGCCCTGCTGTTGTCAGAAAGGAGGAAAAGGTACTTCATGAGTTGTTCGGAACAGACATCAGATGCTGTGCTAGAAATGCACTGGGCCGCGAATGCACCAAGCTGTTCACCATAGATCTAAACCAGGCTCCTCAGAGCACACTGCCCCAGTTATTCCTGAAAGTGGGGGAACCCTTGTGGATCAGGTGTAAGGCCATCCATGTGAACCATGGATTCGGGCTCACCTGGGAGCTGGAAGACAAAGCCCTGGAGGAGGGCAGCTACTTTGAGATGAGTACCTACTCCACAAACAGGACCATGATTCGGATTCTCTTGGCCTTTGTGTCTTCCGTGGGAAGGAACGACACCGGATATTACACCTGCTCTTCCTCAAAGCACCCCAGCCAGTCAGCGTTGGTGACCATCCTAGAAAAAGGGTTTATAAACGCTACCAGCTCGCAAGAAGAGTATGAAATTGACCCGTACGAAAAGTTCTGCTTCTCAGTCAGGTTTAAAGCGTACCCACGAATCCGATGCACGTGGATCTTCTCTCAAGCCTCATTTCCTTGTGAACAGAGAGGCCTGGAGGATGGGTACAGCATATCTAAATTTTGCGATCATAAGAACAAGCCAGGAGAGTACATATTCTATGCAGAAAATGATGACGCCCAGTTCACCAAAATGTTCACGCTGAATATAAGAAAGAAACCTCAAGTGCTAGCAAATGCCTCAGCCAGCCAGGCGTCCTGTTCCTCTGATGGCTACCCGCTACCCTCTTGGACCTGGAAGAAGTGTTCGGACAAATCTCCCAATTGCACGGAGGAAATCCCAGAAGGAGTTTGGAATAAAAAGGCTAACAGAAAAGTGTTTGGCCAGTGGGTGTCGAGCAGTACTCTAAATATGAGTGAGGCCGGGAAAGGGCTTCTGGTCAAATGCTGTGCGTACAATTCTATGGGCACGTCTTGTGAGACGATCCTTTTAAACTCTCCAGGCCCCTTCCCTTTCATCCAAGACAACATCTCATTCTATGCAACAATTGGTGTTTGTCTCCTCTTCATTGTCGTTTTAACCCTGCTAATTTGTCACAAGTACAAAAAGCAATTTAGGTATGAAAGCCAGCTACAGATGGTACAGGTGACCGGCTCCTCAGATAATGAGTACTTCTACGTTGATTTCAGAGAATATGAATATGATCTCAAATATGAATATGATCTCAAATGGGAGTTTCCAAGAGAAAATTTAGAGTTTGGGAAGGTACTAGGATCAGGTGCTTTTGGAAAAGTGATGAACGCAACAGCTTATGGAATTAGCAAAACAGGAGTCTCAATCCAGGTTGCCGTCAAAATGCTGAAAGAAAAAGCAGACAGCTCTGAAAGAGAGGCACTCATGTCAGAACTCAAGATGATGACCCAGCTGGGAAGCCACGAGAATATTGTGAACCTGCTGGGGGCGTGCACACTGTCAGGACCAATTTACTTGATTTTTGAATATTGTTGCTATGGTGACCTCCTCAACTACCTAAGAAGTAAAAGAGAGAAGTTTCACAGGACATGGACAGAGATTTTTAAGGAACATAATTTCAGTTTTTACCCTACTTTCCAGGCACATTCAAATTCCAGCATGCCTGGTTCACGAGAAGTTCAGTTACACCCGCCCTTGGATCAGCTCTCAGGGTTCAATGGGAATTCAATTCATTCTGAAGATGAGATTGAATATGAAAACCAGAAGAGGCTGGCAGAAGAAGAGGAGGAAGATTTGAACGTGCTGACGTTTGAAGACCTCCTTTGCTTTGCGTACCAAGTGGCCAAAGGCATGGAATTCCTGGAGTTCAAGTCGTGTGTCCACAGAGACCTGGCAGCCAGGAATGTGTTGGTCACCCACGGGAAGGTGGTGAAGATCTGTGACTTTGGACTGGCCCGAGACATCCTGAGCGACTCCAGCTACGTCGTCAGGGGCAACGCACGGCTGCCGGTGAAGTGGATGGCACCCGAGAGCTTATTTGAAGGGATCTACACAATCAAGAGTGACGTCTGGTCCTACGGCATCCTTCTCTGGGAGATATTTTCACTGGGTGTGAACCCTTACCCTGGCATTCCTGTCGACGCTAACTTCTATAAACTGATTCAGAGTGGATTTAAAATGGAGCAGCCATTCTATGCCACAGAAGGGATATACTTTGTAATGCAATCCTGCTGGGCTTTTGACTCAAGGAAGCGGCCATCCTTCCCCAACCTGACTTCATTTTTAGGATGTCAGCTGGCAGAGGCAGAAGAAGCGATGTATCAGAACATGGGTGGCAACGTCCCAGAACATCCATCCATCTACCAAAACAGGCGGCCCCTCAGCAGAGAGGCGGGCTCAGAGCCGCCATCGCCACAGGCCCAGGTGAAGATTCACAGAGAAAGAAGTTA

FLT3-TKD p.D835Y

ATGCGGGCGTTGGCGCAGCGCAGCGACCGGCGGCTGCTGCTGCTTGTTGTTTTGTCAGTAATGATTCTTGAGACCGTTACAAACCAAGACCTGCCTGTGATCAAGTGTGTTTTAATCAGTCATGAGAACAATGGCTCATCAGCGGGAAAGCCATCATCGTACCGAATGGTGCGAGGATCCCCAGAAGACCTCCAGTGTGCCCCGAGGCGCCAGAGTGAAGGGACGGTATATGAAGCGGCCACCGTGGAGGTGGCCGAGTCTGGGTCCATCACCCTGCAAGTGCAGCTCGCCACCCCAGGGGACCTTTCCTGCCTCTGGGTCTTTAAGCACAGCTCCCTGGGCTGCCAGCCGCACTTTGATTTACAAAACAGAGGAATCGTTTCCATGGCCATCTTGAACGTGACAGAGACCCAGGCAGGAGAATACCTACTCCATATTCAGAGCGAAGCCGCCAACTACACAGTACTGTTCACAGTGAATGTAAGAGATACACAGCTGTACGTGCTAAGAAGACCTTACTTTAGGAAGATGGAAAACCAGGACGCACTGCTCTGCATCTCCGAGGGTGTTCCAGAGCCCACTGTGGAGTGGGTGCTCTGCAGCTCCCACAGGGAAAGCTGTAAAGAAGAAGGCCCTGCTGTTGTCAGAAAGGAGGAAAAGGTACTTCATGAGTTGTTCGGAACAGACATCAGATGCTGTGCTAGAAATGCACTGGGCCGCGAATGCACCAAGCTGTTCACCATAGATCTAAACCAGGCTCCTCAGAGCACACTGCCCCAGTTATTCCTGAAAGTGGGGGAACCCTTGTGGATCAGGTGTAAGGCCATCCATGTGAACCATGGATTCGGGCTCACCTGGGAGCTGGAAGACAAAGCCCTGGAGGAGGGCAGCTACTTTGAGATGAGTACCTACTCCACAAACAGGACCATGATTCGGATTCTCTTGGCCTTTGTGTCTTCCGTGGGAAGGAACGACACCGGATATTACACCTGCTCTTCCTCAAAGCACCCCAGCCAGTCAGCGTTGGTGACCATCCTAGAAAAAGGGTTTATAAACGCTACCAGCTCGCAAGAAGAGTATGAAATTGACCCGTACGAAAAGTTCTGCTTCTCAGTCAGGTTTAAAGCGTACCCACGAATCCGATGCACGTGGATCTTCTCTCAAGCCTCATTTCCTTGTGAACAGAGAGGCCTGGAGGATGGGTACAGCATATCTAAATTTTGCGATCATAAGAACAAGCCAGGAGAGTACATATTCTATGCAGAAAATGATGACGCCCAGTTCACCAAAATGTTCACGCTGAATATAAGAAAGAAACCTCAAGTGCTAGCAAATGCCTCAGCCAGCCAGGCGTCCTGTTCCTCTGATGGCTACCCGCTACCCTCTTGGACCTGGAAGAAGTGTTCGGACAAATCTCCCAATTGCACGGAGGAAATCCCAGAAGGAGTTTGGAATAAAAAGGCTAACAGAAAAGTGTTTGGCCAGTGGGTGTCGAGCAGTACTCTAAATATGAGTGAGGCCGGGAAAGGGCTTCTGGTCAAATGCTGTGCGTACAATTCTATGGGCACGTCTTGCGAAACCATCTTTTTAAACTCACCAGGCCCCTTCCCTTTCATCCAAGACAACATCTCCTTCTATGCGACCATTGGGCTCTGTCTCCCCTTCATTGTTGTTCTCATTGTGTTGATCTGCCACAAATACAAAAAGCAATTTAGGTACGAGAGTCAGCTGCAGATGATCCAGGTGACTGGCCCCCTGGATAACGAGTACTTCTACGTTGACTTCAGGGACTATGAATATGACCTTAAGTGGGAGTTCCCGAGAGAGAACTTAGAGTTTGGGAAGGTCCTGGGGTCTGGCGCTTTCGGGAGGGTGATGAACGCCACGGCCTATGGCATTAGTAAAACGGGAGTCTCAATTCAGGTGGCGGTGAAGATGCTAAAAGAGAAAGCTGACAGCTGTGAAAAAGAAGCTCTCATGTCGGAGCTCAAAATGATGACCCACCTGGGACACCATGACAACATCGTGAATCTGCTGGGGGCATGCACACTGTCAGGGCCAGTGTACTTGATTTTTGAATATTGTTGCTATGGTGACCTCCTCAACTACCTAAGAAGTAAAAGAGAGAAGTTTCACAGGACATGGACAGAGATTTTTAAGGAACATAATTTCAGTTTTTACCCTACTTTCCAGGCACATTCAAATTCCAGCATGCCTGGTTCACGAGAAGTTCAGTTACACCCGCCCTTGGATCAGCTCTCAGGGTTCAATGGGAACTCAATTCATTCTGAAGATGAGATTGAATATGAAAACCAGAAGAGGCTGGCAGAAGAAGAGGAGGAAGATTTGAACGTGCTGACGTTTGAAGACCTCCTTTGCTTTGCGTACCAAGTGGCCAAAGGCATGGAGTTCCTGGAGTTCAAGTCGTGTGTCCACAGAGACCTGGCAGCCAGGAATGTGTTGGTCACCCACGGGAAGGTGGTGAAGATCTGTGACTTTGGGCTAGCCCGATACATCCTGAGCGACTCCAGCTACGTCGTCAGGGGCAACGCACGGCTGCCGGTGAAGTGGATGGCACCCGAGAGCTTATTTGAAGGGATCTACACAATCAAGAGTGACGTCTGGTCCTACGGCATCCTTCTCTGGGAGATATTTTCACTGGGTGTGAACCCTTACCCTGGCATTCCTGTCGACGCTAACTTCTATAAACTGATTCAGAGTGGATTTAAAATGGAGCAGCCATTCTATGCCACAGAAGGGATATACTTTGTAATGCAATCCTGCTGGGCTTTTGACTCAAGGAAGCGGCCATCCTTCCCCAACCTGACTTCATTTTTAGGATGTCAGCTGGCAGAGGCAGAAGAAGCGATGTATCAGAACATGGGTGGCAACGTCCCAGAACATCCATCCATCTACCAAAACAGGCGGCCCCTCAGCAGAGAGGCGGGCTCAGAGCCGCCATCGCCACAGGCCCAGGTGAAGATTCACAGAGAAAGAagttaa

NPM1::ALK

atggaagattcgatggacatggacatgagccccctgaggccccagaactatcttttcggttgtgaactaaaggccgacaaagattatcactttaaggtggataatgatgaaaatgagcaccagttatctttaagaacggtcagtttaggggctggtgcaaaggatgagttgcacattgttgaagcagaggcaatgaattacgaaggcagtccaattaaagtaacactggcaactttgaaaatgtctgtacagccaacggtttcccttgggggctttgaaataacaccaccagtggtcttaaggttgaagtgtggttcagggccagtgcatattagtggacagcacttagtagtgtaccgccggaagcaccaggagctgcaagccatgcagatggagctgcagagccctgagtacaagctgagcaagctccgcacctcgaccatcatgaccgactacaaccccaactactgctttgctggcaagacctcctccatcagtgacctgaaggaggtgccgcggaaaaacatcaccctcattcggggtctgggccatggcgcctttggggaggtgtatgaaggccaggtgtccggaatgcccaacgacccaagccccctgcaagtggctgtgaagacgctgcctgaagtgtgctctgaacaggacgaactggatttcctcatggaagccctgatcatcagcaaattcaaccaccagaacattgttcgctgcattggggtgagcctgcaatccctgccccggttcatcctgctggagctcatggcggggggagacctcaagtccttcctccgagagacccgccctcgcccgagccagccctcctccctggccatgctggaccttctgcacgtggctcgggacattgcctgtggctgtcagtatttggaggaaaaccacttcatccaccgagacattgctgccagaaactgcctcttgacctgtccaggccctggaagagtggccaagattggagacttcgggatggcccgagacatctacagggcgagctactatagaaagggaggctgtgccatgctgccagttaagtggatgcccccagaggccttcatggaaggaatattcacttctaaaacagacacatggtcctttggagtgctgctatgggaaatcttttctcttggatatatgccataccccagcaaaagcaaccaggaagttctggagtttgtcaccagtggaggccggatggacccacccaagaactgccctgggcctgtataccggataatgactcagtgctggcaacatcagcctgaagacaggcccaactttgccatcattttggagaggattgaatactgcacccaggacccggatgtaatcaacaccgctttgccgatagaatatggtccacttgtggaagaggaagagaaagtgcctgtgaggcccaaggaccctgagggggttcctcctctcctggtctctcaacaggcaaaacgggaggaggagcgcagcccagctgccccaccacctctgcctaccacctcctctggcaaggctgcaaagaaacccacagctgcagaggtctctgttcgagtccctagagggccggccgtggaagggggacacgtgaatatggcattctctcagtccaaccctccttcggagttgcacaaggtccacggatccagaaacaagcccaccagcttgtggaacccaacgtacggctcctggtttacagagaaacccaccaaaaagaataatcctatagcaaagaaggagccacacgacaggggtaacctggggctggagggaagctgtactgtcccacctaacgttgcaactgggagacttccgggggcctcactgctcctagagccctcttcgctgactgccaatatgaaggaggtacctctgttcaggctacgtcacttcccttgtgggaatgtcaattacggctaccagcaacagggcttgcccttagaagccgctactgcccctggagctggtcattacgaggataccattctgaaaagcaagaatagcatgaaccagcctgggccctga

KMT2A::MLLT3

ggcaattccgcgaacatggcgcacagctgtcggtggcgcttccccgcccgacccgggaccaccgggggcggcggcggcggggggcgccggggcctagggggcgccccgcggcaacgcgtcccggccctgctgcttccccccgggcccccggtcggcggtggcggccccggggcgcccccctcccccccggctgtggcggccgcggcggcggcggcgggaagcagcggggctggggttccagggggagcggccgccgcctcagcagcctcctcgtcgtccgcctcgtcttcgtcttcgtcatcgtcctcagcctcttcagggccggccctgctccgggtgggcccgggcttcgacgcggcgctgcaggtctcggccgccatcggcaccaacctgcgccggttccgggccgtgtttggggagagcggcgggggaggcggcagcggagaggatgagcaattcttaggttttggctcagatgaagaagtcagagtgcgaagtcccacaaggtctccttcagttaaaactagtcctcgaaaacctcgtgggagacctagaagtggctctgaccgaaattcagctatcctctcagatccatctgtgttttcccctctaaataaatcagagaccaaatctggagataagatcaagaagaaagattctaaaagtatagaaaagaagagaggaagacctcccaccttccctggagtaaaaatcaaaataacacatggaaaggacatttcagagttaccaaagggaaacaaagaagatagcctgaaaaaaattaaaaggacaccttctgctacgtttcagcaagccacaaagattaaaaaattaagagcaggtaaactctctcctctcaagtctaagtttaagacagggaagcttcaaataggaaggaagggggtacaaattgtacgacggagaggaaggcctccatcaacagaaaggataaagaccccttcgggtctcctcattaattctgaactggaaaagccccagaaagtccggaaagacaaggaaggaacacctccacttacaaaagaagataagacagttgtcagacaaagccctcgaaggattaagccagttaggattattccttcttcaaaaaggacagatgcaaccattgctaagcaactcttacagagggcaaaaaagggggctcaaaagaaaattgaaaaagaagcagctcagctgcagggaagaaaggtgaagacacaggtcaaaaatattcgacagttcatcatgcctgttgtcagtgctatctcctcgcggatcattaagacccctcggcggtttatagaggatgaggattatgaccctccaattaaaattgcccgattagagtctacaccgaatagtagattcagtgccccgtcctgtggatcttctgaaaaatcaagtgcagcttctcagcactcctctcaaatgtcttcagactcctctcgatctagtagccccagtgttgatacctccacagactctcaggcttctgaggagattcaggtacttcctgaggagcggagcgatacccctgaagttcatcctccactgcccatttcccagtccccagaaaatgagagtaatgataggagaagcagaaggtattcagtgtcggagagaagttttggatctagaacgacgaaaaaattatcaactctacaaagtgccccccagcagcagacctcctcgtctccacctccacctctgctgactccaccgccaccactgcagccagcctccagtatctctgaccacacaccttggcttatgcctccaacaatccccttagcatcaccatttttgcctgcttccactgctcctatgcaagggaagcgaaaatctattttgcgagaaccgacatttaggtggacttctttaaagcattctaggtcagagccacaatacttttcctcagcaaagtatgccaaagaaggtcttattcgcaaaccaatatttgataatttccgaccccctccactaactcccgaggacgttggctttgcatctggtttttctgcatctggtaccgctgcttcagcccgattgttttcgccactccattctggaacaaggtttgatatgcacaaaaggagccctcttctgagagctccaagatttactccaagtgaggctcactctagaatatttgagtctgtaaccttgcctagtaatcgaacttctgctggaacatcttcttcaggagtatccaatagaaaaaggaaaagaaaagtgtttagtcctattcgatctgaaccaagatctccttctcactccatgaggacaagaagtggaaggcttagtagttctgagctctcacctctcacccccccgtcttctgtctcttcctcgttaagcatttctgttagtcctcttgccactagtgccttaaacccaacttttacttttccttctcattccctgactcagtctggggaatctgcagagaaaaatcagagaccaaggaagcagactagtgctccggcagagccattttcatcaagtagtcctactcctctcttcccttggtttaccccaggctctcagactgaaagagggagaaataaagacaaggcccccgaggagctgtccaaagatcgagatgctgacaagagcgtggagaaggacaagagtagagagagagaccgggagagagaaaaggagaataagcgggagtcaaggaaagagaaaaggaaaaagggatcagaaattcagagtagttctgctttgtatcctgtgggtagggtttccaaagagaaggttgttggtgaagatgttgccacttcatcttctgccaaaaaagcaacagggcggaagaagtcttcatcacatgattctgggactgatattacttctgtgactcttggggatacaacagctgtcaaaaccaaaatacttataaagaaagggagaggaaatctggaaaaaaccaacttggacctcggcccaactgccccatccctggagaaggagaaaaccctctgcctttccactccttcatctagcactgttaaacattccacttcctccataggctccatgttggctcaggcagacaagcttccaatgactgacaagagggttgccagcctcctaaaaaaggccaaagctcagctctgcaagattgagaagagtaagagtcttaaacaaaccgaccagcccaaagcacagggtcaagaaagtgactcatcagagacctctgtgcgaggaccccggattaaacatgtctgcagaagagcagctgttgcccttggccgaaaacgagctgtgtttcctgatgacatgcccaccctgagtgccttaccatgggaagaacgagaaaagattttgtcttccatggggaatgatgacaagtcatcaattgctggctcagaagatgctgaacctcttgctccacccatcaaaccaattaaacctgtcactagaaacaaggcaccccaggaacctccagtaaagaaaggacgtcgatcgaggcggtgtgggcagtgtcccggctgccaggtgcctgaggactgtggtgtttgtactaattgcttagataagcccaagtttggtggtcgcaatataaagaagcagtgctgcaagatgagaaaatgtcagaatctacaatggatgccttccaaagcctacctgcagaagcaagctaaagctgtgaaaaagaaagagaaaaagtctaagaccagtgaaaagaaagacagcaaagagagcagtgttgtgaagaacgtggtggactctagtcagaaacctaccccatcagcaagagaggatcctgccccaaagaaaagcagtagtgagcctcctccacgaaagcccgtcgaggaaaagagtgaagaagggaatgtctcggcccctgggcctgaatccaaacaggccaccactccagcttccaggaagtcaagcaagcaggtctcccagccagcactggtcatcccgcctcagccacctactacaggaccgccaagaaaagaagttcccaaaaccactcctagtgagcccaagaaaaagcagcctccaccaccagaatcaggtccagagcagagcaaacagaaaaaagtggctccccgcccaagtatccctgtaaaacaaaaaccaaaagaaaaggaaaaaccacctccggtcaataagcaggagaatgcaggcactttgaacatcctcagcactctctccaatggcaatagttctaagcaaaaaattccagcagatgggtcgcgaagtatacacacgtgtcttgaagtgaaaagtccaataaagcaaagcaaatcagataagcaaataaagaatggtgaatgtgacaaggcatacctagatgaactggtagagcttcacagaaggttaatgacattgagagaaagacacattctgcagcagatcgtgaaccttatagaagaaactggacactttcatatcacaaacacaacatttgattttgatctttgctcgctggacaaaaccacagtccgtaaactacagagttacctggaaacatctggaacatcctga

**Data S1**

Uncropped version of immunoblot depicted in Supplementary Fig. S11**a**

Gel


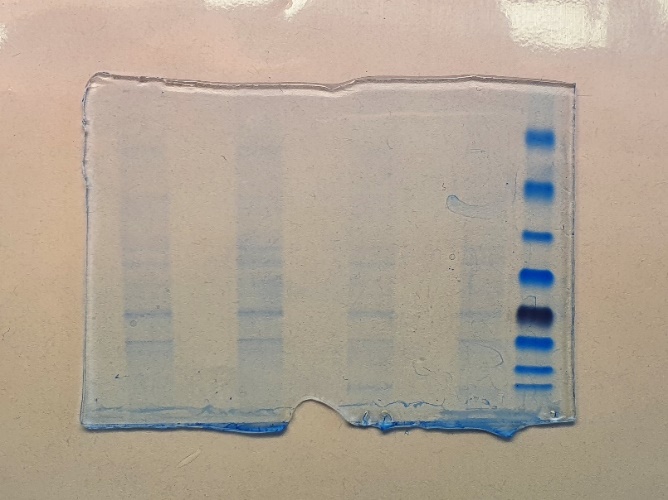


Anti-HA-antibody


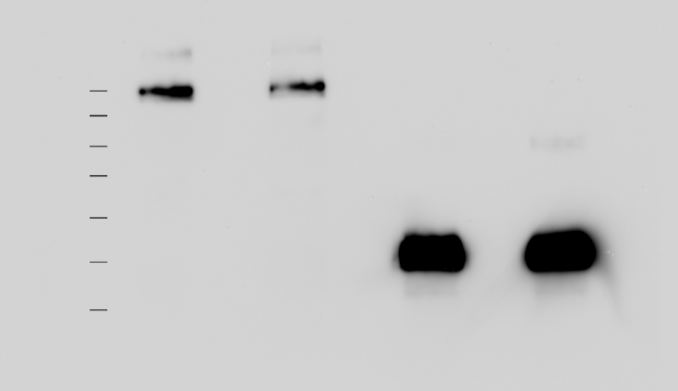


Marker


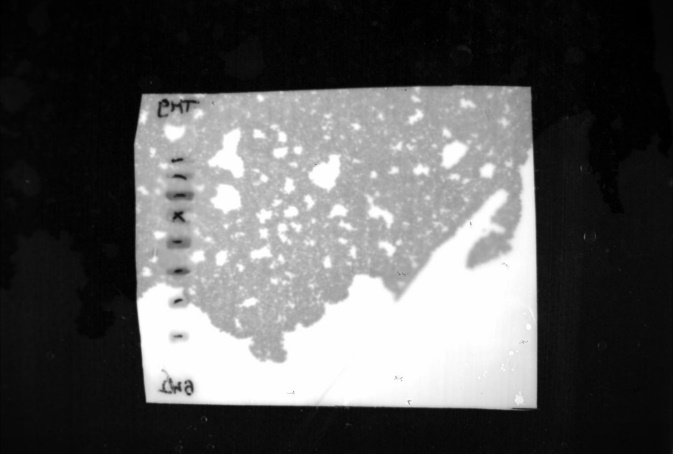

Supplement: Supplementary file 1 — Supplementary Data [file 41392_2025_2491_MOESM1_ESM.docx]
